# Combining Neprilysin Inhibitor With AT_2_R Agonist Is Superior to Combination With AT_1_R Blocker in Providing Reno-Protection in Obese Rats

**DOI:** 10.3389/fphar.2021.778953

**Published:** 2022-02-07

**Authors:** Elizabeth Alana Gray, Sanket N. Patel, Peter A. Doris, Tahir Hussain

**Affiliations:** ^1^ Department of Pharmacological and Pharmaceutical Sciences, College of Pharmacy, University of Houston, Houston, TX, United States; ^2^ The Brown Foundation Institute of Molecular Medicine Center for Human Genetics, The University of Texas Health Science Center at Houston, Houston, TX, United States

**Keywords:** angiotensin receptor blocker, angiotensin II type 2 receptor agonist, neprilysin inhibition, reno-protection, proteinuria, obesity

## Abstract

Clinical use of the combination therapy of the neprilysin inhibitor sacubitril and angiotensin II type 1 receptor blocker valsartan is known to be associated with albuminuria. Albuminuria is both a risk factor for and an indicator of kidney injury. Earlier work from our laboratory reported that the agonist of angiotensin II type 2 receptor Compound 21 (C21) prevents proteinuria, albuminuria, and is reno-protective in obese Zucker rats fed high salt diet (HSD). Thus, we hypothesized that sacubitril/C21 combination provides superior reno-protection compared to sacubitril/valsartan. Male obese Zucker rats 10–11 weeks old were treated daily with vehicle, sacubitril + C21, or sacubitril + valsartan while fed HSD for 16 days. HSD-feeding caused kidney dysfunction, evident by significant increases in urinary protein, osteopontin, and cystatin C. HSD-feeding lowered plasma cystatin C and creatinine concentrations suggestive of hyperfiltration, which was not affected by either treatment. Unlike sacubitril/valsartan, sacubitril/C21 treatment significantly decreases proteinuria, albuminuria, the expression of nephrin, and kidney weight, independent of hyperfiltration, compared with HSD alone. Moreover, sacubitril/valsartan therapy increased plasma renin and did not prevent HSD-induced increases in renal angiotensin II, while sacubitril/C21 completely prevented these changes. Together, this study suggests that sacubitril/C21 afforded superior reno-protection compared to sacubitril/valsartan therapy in high salt-fed obese Zucker rats.

## Introduction

Prevalence of obesity, a serious and costly condition has been increasing steadily over the past 30 years. With obesity, an abnormally regulated renin angiotensin system (RAS) contributes to the onset and progression of kidney damage, establishing a link between obesity and kidney dysfunction (Hall et al., 2020). While obesity is an independent risk factor for kidney diseases (Rutkowski et al., 2006), obesity is a gateway disease that increases the risk of developing hypertension and diabetes, two leading causes of end-stage renal disease (Maric-Bilkan, 2013). Hypertension and diabetes are similarly associated with an abnormally regulated RAS (Yim and Yoo, 2008), in which renal concentrations of angiotensin II (Ang II) and angiotensin II type 1 receptor (AT_1_R) function are elevated, and levels of reno-protective angiotensin-(1–7) [Ang-(1–7)] and natriuretic peptides are decreased, along with angiotensin-converting enzyme 2 (ACE2) expression and activity (Tikellis et al., 2012). Together, these changes accelerate kidney injury via their anti-diuretic, anti-natriuretic, and pro-inflammatory effects. Thus, the co-existence of obesity and these common co-morbidities increases the risk of functional and structural damage to the kidney (Schiffrin et al., 2007). For these reasons, clinically used anti-hypertensive mainstay therapies that provide reno-protection and reduce the risk of progression to end-stage renal disease including angiotensin converting enzyme (ACE) inhibitors and angiotensin receptor blockers (ARBs) were developed (Wenzel, 2005). Despite the availability and use of these treatments, patients remain at risk of worsening kidney injury (Haynes et al., 2018) and some require combination therapy to control blood pressure (Wenzel, 2005; Shimosawa, 2013; Helmer et al., 2018).

In addition to reno-protective Ang-(1–7) and it’s generating enzymes ACE2 and neprilysin, angiotensin II type 2 receptor (AT_2_R) and receptor Mas are similarly classified as components of the alternative RAS axis. The actions of the alternative RAS axis oppose those of the classical RAS axis, which includes Ang II and AT_1_R. This knowledge led to the identification of novel RAS targets, such as AT_2_R. Developed by Vicore Pharma, the agonist of AT_2_R Compound 21 (C21), is a non-peptide agonist that binds selectively to the receptor, activating the nitric oxide-cyclic guanosine monophosphate pathway (Matavelli and Siragy, 2015; Pandey and Gaikwad, 2017). Currently in clinical trials for pulmonary fibrosis, C21 represents a class of drugs with strong potential for clinical use (Matavelli and Siragy, 2015; George et al., 2020). Furthermore, recent crystallization of the AT_2_R, has revealed its unique structural conformations, explaining why the receptor does not undergo rapid desensitization or internalization following agonist exposure (Zhang et al., 2017), adding to the support of AT_2_R as a target for drug therapy. While multiple studies using different animal models have similarly concluded that C21 treatment is associated with reno- and tissue-protective effects, in agreement our laboratory has previously demonstrated that pharmacological activation of AT_2_R in obese rats by C21 has blood pressure lowering, diuretic, natriuretic, and anti-inflammatory/-oxidant effects (Ali and Hussain, 2012; Koulis et al., 2015).

Natriuretic peptides including atrial natriuretic peptide (ANP) similarly drive diuresis and natriuresis and are known to lower blood pressure (Haynes et al., 2018). The beneficial effects of preventing the enzymatic breakdown of ANP with inhibitors of neprilysin has been demonstrated (Helin et al., 1991). However, neprilysin inhibition via reflex RAS activation can lead to increases in Ang II concentrations (Richards et al., 1993). Therefore, a combination therapy of a neprilysin inhibitor with an ARB to selectively block the interaction between Ang II and AT_1_R was developed. Subsequent to its rapid approval, post-hoc studies focused on the renal effects of the combined neprilysin inhibitor and ARB uncovered that compared to enalapril treatment, while the combination slowed the rate of decline in estimated glomerular filtration rate (eGFR), the urinary albumin to creatinine ratio was significantly increased (Damman et al., 2018). The knowledge that the ratio of urinary albumin to creatinine, a prognostic marker used to improve the prediction of end-stage renal disease was increased, and chronic treatment with C21 in obese Zucker rats (OZR) prevented albuminuria (Patel et al., 2016), taken together, led to the hypothesis that combination therapy with the neprilysin inhibitor sacubitril (SAC) and AT_2_R agonist C21 will provide greater reno-protection as compared to the neprilysin inhibitor combined with the AT_1_R blocker valsartan (VAL). Therefore, the goal of this study was to investigate and compare the kidney-specific outcomes of the current SAC/VAL therapy to our novel approach of SAC/C21 in chronically treated OZR fed a high salt diet (HSD). Outcomes from this study suggest that C21 in combination with neprilysin inhibition is superior in preventing decline in kidney function, albuminuria, and proteinuria, as well as in reversing the changes in Ang II and renin, while similarly preserving ANP.

## Methods

### Animals

Male obese Zucker rats (OZR), 10–11 weeks of age, were purchased from Envigo in Indianapolis. Following arrival, the rats were housed in the animal care facility at University of Houston. Experimental protocols were approved by the IACUC at the University of Houston (protocol number 15-035) and were conducted in accordance with the NIH Care and Use of Laboratory Animal Guidelines. All rats were acclimatized for 7–10 days, prior to beginning any treatment. Simultaneously on the day that treatment began, all rats were also placed onto a 0.4% normal salt diet (NSD) (Teklad custom diet TD.99215, Envigo Teklad Diet Madison, WI, United States) or 4% high salt diet (HSD) (Teklad custom diet TD 92034, Envigo Teklad Diets Madison, WI, United States). Based on prior publications from our laboratory, 2 weeks of HSD-feeding in OZR resulted in kidney functional injury, including proteinuria (Patel et al., 2016). Therefore, treatments were delivered daily for 16 days via oral gavage at the same time each day and consisted of either vehicle (20 μl DMSO & 580 μl corn oil), AT_2_R agonist C21, 1 mg/kg/day (a gift from Vicore Pharma) & sacubitril, 10 mg/kg/day (Cayman Chemical company Item 21473) or valsartan, 10 mg/kg/day (Cayman chemical company 14178) & sacubitril, 10 mg/kg/day. Thus, there were four groups of OZRs which each consisted of an *n* = 8; NSD + vehicle (NSD), HSD + vehicle (HSD), HSD + sacubitril/C21 (SAC/C21), and HSD + sacubitril/valsartan (SAC/VAL). Dosages for SAC and VAL in combination, and for C21 were chosen based on prior publications (Ali et al., 2015; Kusaka et al., 2015). Urine was collected from individually housed rats over a 24 h time frame on day 13 via metabolic cages. The metal wired floor of each metabolic cage was connected to a funnel that allowed for urine to be collected separately from feces into 50 ml tubes containing mineral oil to prevent loss of urine over the duration of urine collection. Food and water intake, and body weight were measured every other day. All animals were euthanized under isoflurane, and blood for plasma collection was acquired via cardiac puncture and collected in EDTA coated tubes containing a mixture of protease and phosphatase inhibitors. Blood samples were spun at 1,000 g for 30 min at 4°C for collection of plasma, which was aliquoted and stored at −80°C, along with all urine and tissue samples which were snap frozen in liquid nitrogen immediately after collection. For detailed methods, please refer to the Supplementary Material.

### Statistical Analysis

GraphPad Prism Version 8.4.3 was used for data analysis via one-way ANOVA with Fisher’s LSD test. Data is presented as mean ± SEM, *p < 0.05, **p < 0.01, ****p < 0.0001; *n* = 6–8 per group.

## Results

### General Parameters

In comparison to OZR fed NSD, those on HSD drank significantly more water over the 16 days study period (NSD 366 ± 15 ml vs HSD 742 ± 15 ml), which resulted in an increased daily urine volume (NSD 8.8 ± 1.4 ml/24 h vs HSD 28.4 ± 2.6 ml/24 h). Though water intake was not impacted by either treatment, SAC/VAL treated OZR produced modestly less urine volume over a 24 h period. HSD-fed OZR took in less food over the duration of the study compared to the NSD-fed OZR (HSD 394 ± 11 g vs NSD 517 ± 38 g), neither treatment affected total food intake. While initial body weight did not differ between the groups, one interesting outcome was that SAC/C21 treatment significantly prevented weight gain (HSD 57.6 ± 6.8 g vs SAC/C21 37.3 ± 3.9 g) (Table 1).

**TABLE 1:** General parameters.

	NSD	HSD	SAC/C21	SAC/VAL
Day 1 body weight (g)	459 ± 8.6	485 ± 9.8	474 ± 13.88	484 ± 10.25
Day 16 body weight (g)	526.5 ± 9.8	542.6 ± 12.1	510.1 ± 14.9	529.6 ± 10.9
Weight gain (g)	67.3 ± 8.0	57.6 ± 6.8	37.3 ± 3.9^†^	45.4 ± 7.6
Total food intake (g)	517.0 ± 37.9	394.1 ± 11.1*	367.6 ± 4.4	369.3 ± 10.5
Total water intake (ml)	366.1 ± 15.1	741.8 ± 15.0*	718.8 ± 34.5	677.6 ± 25.3
24 h urine vol. (ml)	8.8 ± 1.4	28.4 ± 2.6*	22.6 ± 2.5	20.7 ± 2.3^†^
Plasma sodium (mg/L)	3015 ± 58.0	2886 ± 77.0	2982 ± 112.2	2880 ± 62.3

Data shown as mean ± SEM, analyzed by one-way ANOVA, followed by Fisher’s LSD test. Results are considered significant at *p < 0.05 and ^†^ *p* < 0.05 with a 95% confidence interval; *significantly different from NSD, ^†^significantly different from HSD; *n* = 8 for all parameters.

### Kidney Function

**FIGURE 1:**
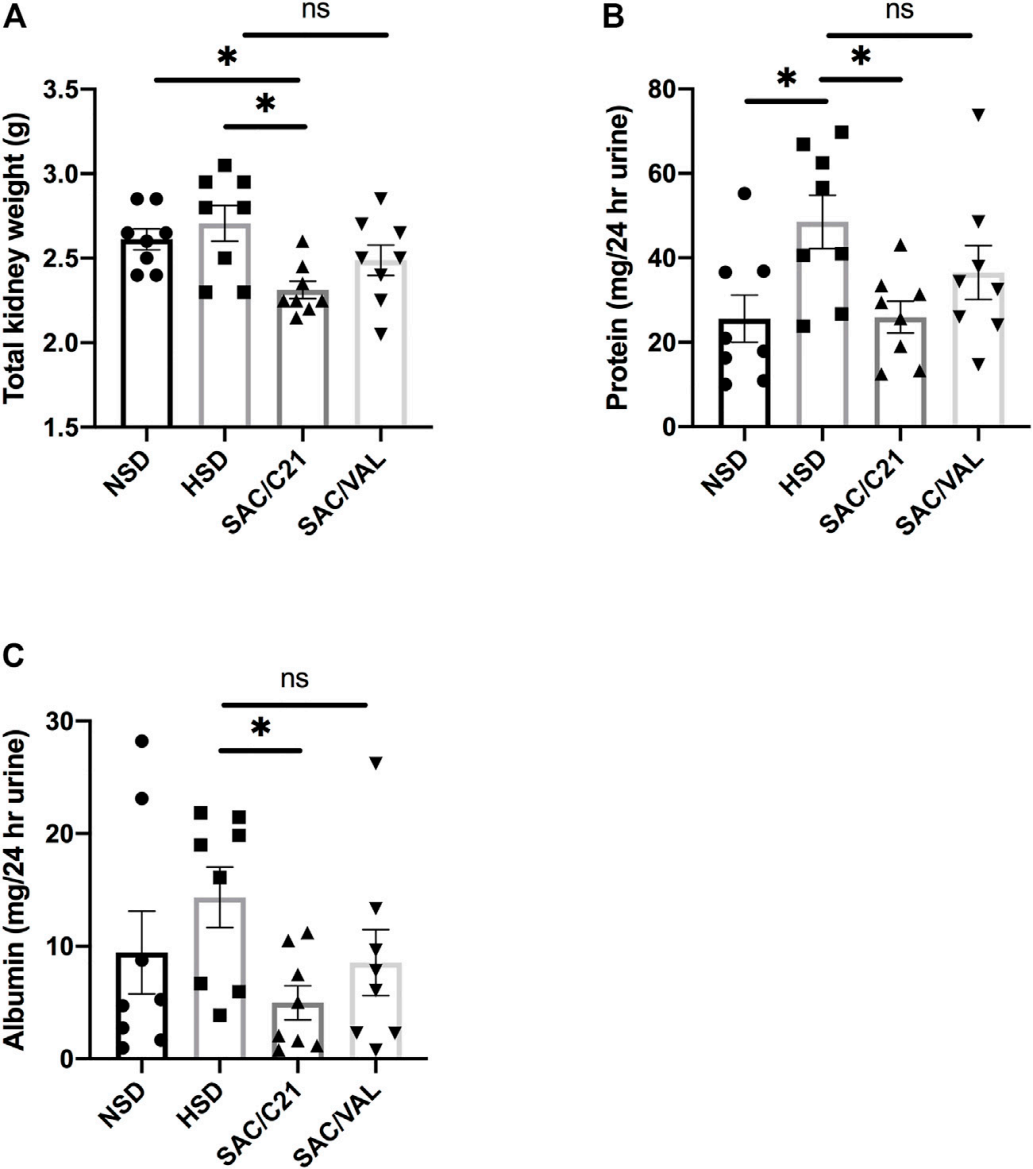
**(A)** kidney weight, **(B)** urinary protein normalized to 24 h urine volume, **(C)** urinary albumin normalized to 24 h urine volume in obese Zucker rats on NSD or HSD and treated with vehicle, SAC/C21, or SAC/VAL. Data shown as mean ± SEM, analyzed by one-way ANOVA, followed by Fisher’s LSD test. Results are considered significant at *p < 0.05, with a 95% confidence interval; *n* = 8. NSD—normal salt diet fed + vehicle treated obese control, HSD—high salt diet fed + vehicle treated obese control, SAC/C21—high salt diet fed obese rat treated with sacubitril + C21, SAC/VAL—high salt diet fed obese rat treated with sacubitril + valsartan.

Kidney weight at the time of euthanasia in SAC/C21 treatment group was lower compared to that of HSD-fed rats (HSD 2.7 ± 0.1 g vs SAC/C21 2.3 ± 0.05 g) (Figure 1A). Total urinary protein and urinary albumin measurements revealed that HSD-feeding increased proteinuria and albuminuria. Combination treatment with SAC/C21 provided protection against increases in proteinuria and albuminuria (HSD 48.5 ± 6.3 vs SAC/C21 25.9 ± 3.7 mg protein/24 h urine, and HSD 14.3 ± 2.7 vs SAC/C21 4.9 ± 1.5 mg albumin/24 h urine, respectively) (Figures 1B,C). SAC/VAL treatment did not reduce HSD-induced proteinuria and albuminuria.

### Markers of Kidney Damage

**FIGURE 2:**
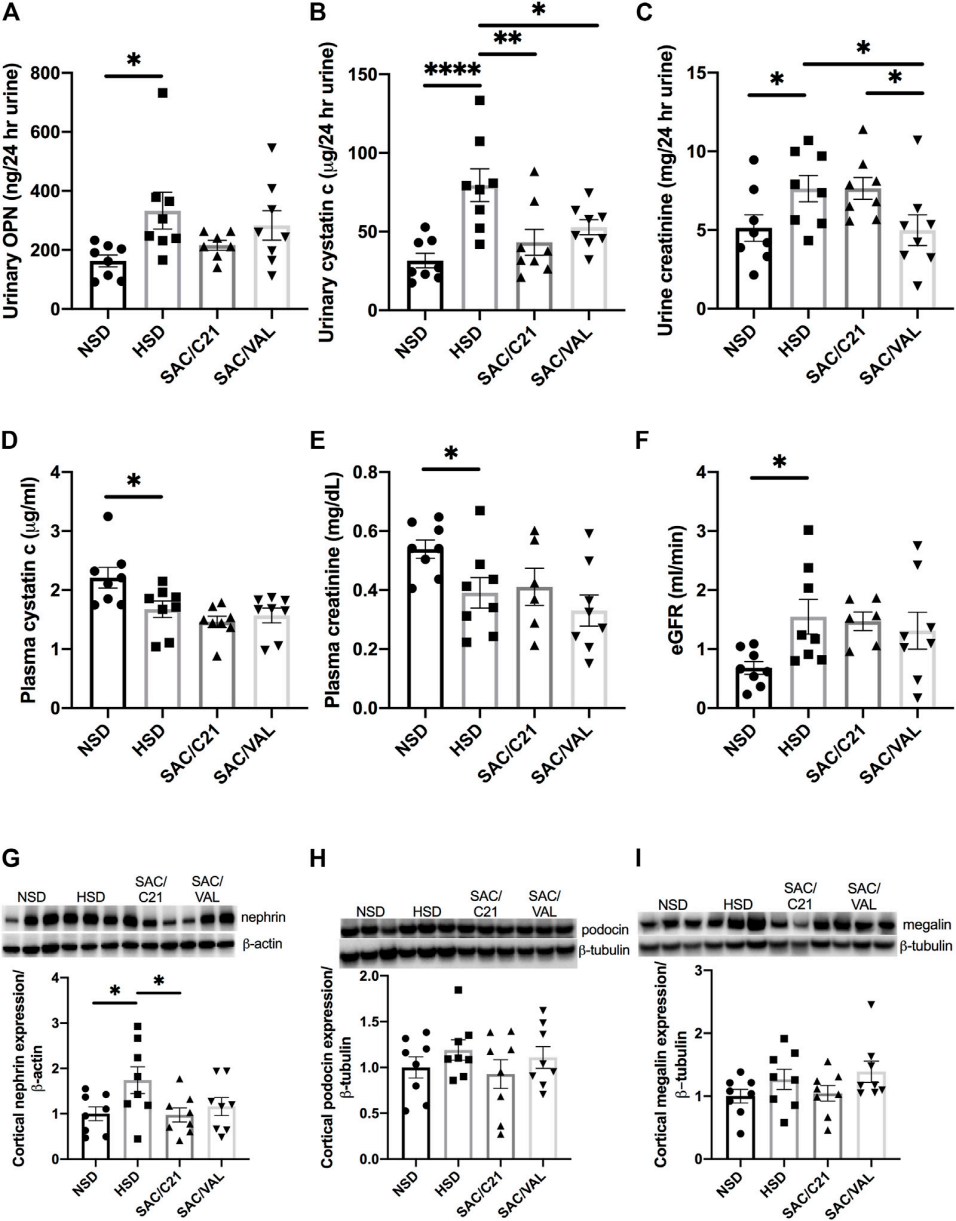
Analysis of renal injury via markers and indicators of glomerular and tubular damage **(A)** urinary osteopontin normalized to 24 h urine volume, **(B)** urinary cystatin C normalized to 24 h urine volume, **(C)** urinary creatinine, **(D)** plasma cystatin C, **(E)** plasma creatinine, **(F)** eGFR, **(G)** nephrin expression in kidney cortex, **(H)** podocin expression in kidney cortex, and **(I)** megalin expression in kidney cortex from obese Zucker rats on NSD or HSD and treated with vehicle, SAC/C21, or SAC/VAL. Data shown as mean ± SEM, analyzed by one-way ANOVA, followed by Fisher’s LSD test. Results are considered significant at *p < 0.05, **p < 0.01, ****p < 0.0001 with a 95% confidence interval; *n* = 6–8 for urinary osteopontin, plasma creatinine, and eGFR and *n* = 8 for urinary and plasma cystatin C, urinary creatinine, nephrin, podocin, and megalin expression. NSD—normal salt diet fed + vehicle treated obese control, HSD—high salt diet fed + vehicle treated obese control, SAC/C21—high salt diet fed obese rat treated with sacubitril + C21, SAC/VAL—high salt diet fed obese rat treated with sacubitril + valsartan.

Urinary concentrations of osteopontin, a marker used to predict incident chronic kidney disease (CKD) were normalized to 24 h urine volume and determined to be increased by HSD-feeding and modestly decreased by treatment with SAC/C21 (NSD 163 ± 19.8 vs HSD 333.3 ± 62.3 vs SAC/C21 216.1 ± 17 ng/24 h urine) (Figure 2A). Urinary cystatin C was similarly increased by HSD-feeding, a response that was inhibited by both treatments (HSD 79.5 ± 10.4 vs SAC/C21 43.23 ± 8.2 vs SAC/VAL 52.8 ± 4.7 µg/24 h urine) (Figure 2B). Urine creatinine excretion was significantly increased by HSD-feeding and lowered by SAC/VAL (HSD 7.6 ± 0.8 vs SAC/VAL 5.0 ± 0.9 mg/24 h urine) (Figure 2C). Measurements of plasma cystatin C and creatinine revealed that both were lowered by HSD-feeding (NSD 2.2 ± 0.17 vs HSD 1.7 ± 0.14 μg/ml and NSD 0.54 ± 0.03 vs HSD 0.39 ± 0.05 mg/dl) (Figures 2D,E respectively). Calculation of eGFR showed it to be increased by HSD-feeding for 16 days (NSD 0.7 ± 0.1 vs HSD 1.5 ± 0.3 ml/min) (Figure 2F). Western blot analysis on the expression of two slit diaphragm proteins, nephrin and podocin suggest that similarly only SAC/C21 treatment limited damage to the glomerular filtration barrier as evidenced by the lack of nephrin upregulation compared to HSD-fed rats (HSD 1.7 ± 0.3 vs SAC/C21 0.97 ± 0.15) (Figure 2G). The expression of podocin and megalin, a proximal tubule transporter were not significantly different among the four groups (Figures 2H,I).

### The Activity of Neprilysin and ACE2, Expression of Neprilysin, ACE2, and NPR-C and Plasma ANP and Bradykinin

**FIGURE 3:**
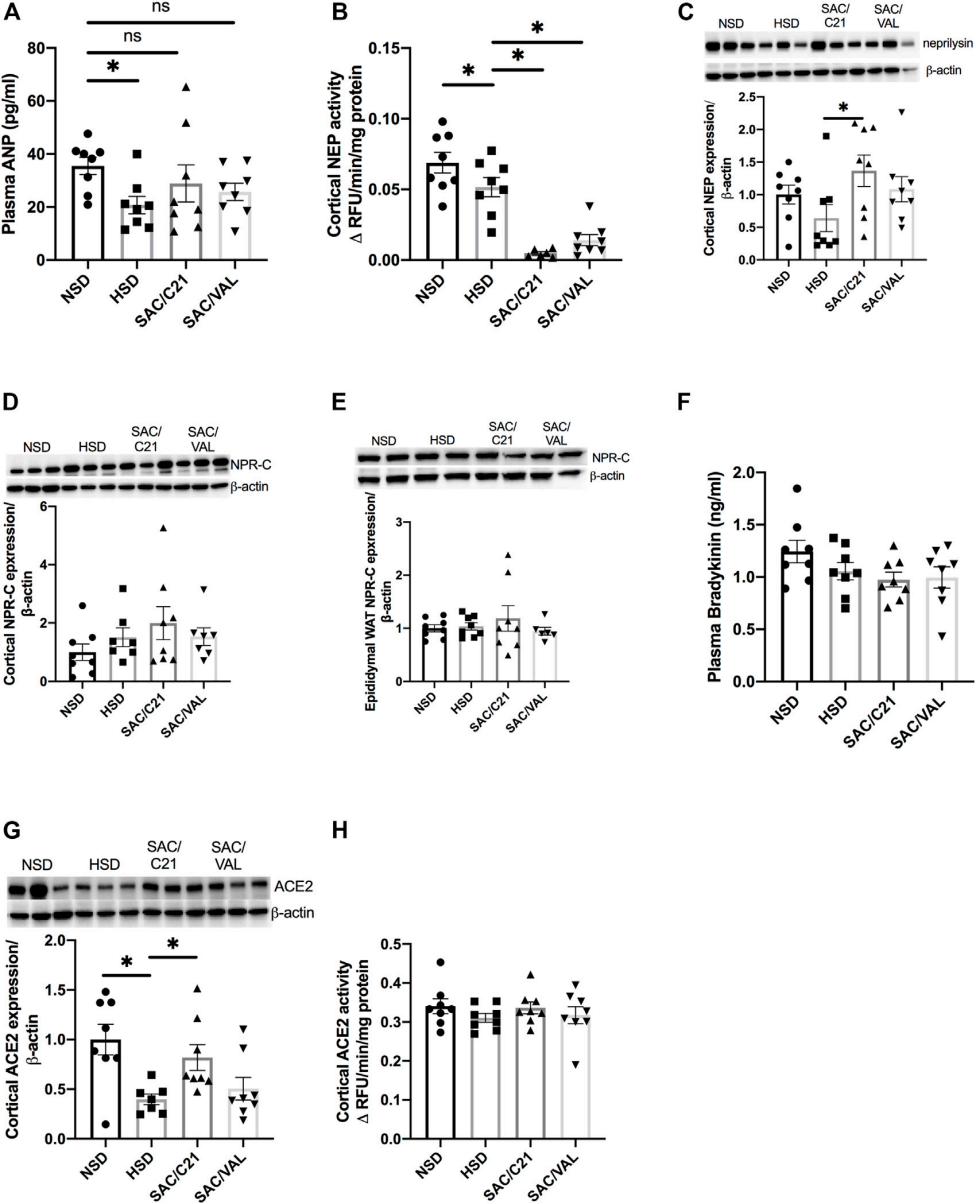
**(A)** plasma ANP, **(B)** Neprilysin activity in kidney cortex, **(C)** Neprilysin expression in kidney cortex, **(D)** NPR-C expression in kidney cortex, **(E)** NPR-C expression in white adipose tissue, **(F)** plasma bradykinin, **(G)** ACE2 expression in kidney cortex, and **(H)** ACE2 activity in kidney cortex from obese Zucker rats on NSD or HSD and treated with vehicle, SAC/C21, or SAC/VAL. Data shown as mean ± SEM, analyzed by one-way ANOVA, followed by Fisher’s LSD test. Results are considered significant at *p < 0.05, with a 95% confidence interval; *n* = 6–8 for neprilysin activity and renal NPR-C and ACE2 expression and *n* = 8 for plasma ANP and bradykinin, expression of renal neprilysin and adipose NPR-C, and renal ACE2 activity. NSD—normal salt diet fed + vehicle treated obese control, HSD—high salt diet fed + vehicle treated obese control, SAC/C21—high salt diet fed obese rat treated with sacubitril + C21, SAC/VAL—high salt diet fed obese rat treated with sacubitril + valsartan.

Circulating ANP was decreased by HSD-feeding, but the treatments preserved plasma levels of ANP (Figure 3A). Renal activity of neprilysin was significantly decreased in the kidney of both treated groups compared with HSD-fed control OZR (HSD 0.05 ± 0.007 vs SAC/C21 0.005 ± 0.001 and SAC/VAL 0.01 ± 0.004) (Figure 3B). Western blot measurement of neprilysin expression revealed no change by HSD-feeding, but there was a significant increase in SAC/C21 treated OZR (HSD 0.63 ± 0.2 vs SAC/C21 1.4 ± 0.2) (Figure 3C). Expression of the clearance natriuretic peptide receptor type C (NPR-C) in the kidney cortex was modestly increased by HSD-feeding (NSD 1.0 ± 0.3 vs. HSD 1.5 ± 0.3), but unchanged in epididymal white adipose tissue (Figures 3D,E). Circulating levels of bradykinin (BK) remained unchanged among all groups and were not elevated by SAC/C21 treatment (Figure 3F). The expression of ACE2 was significantly decreased in HSD-fed OZR compared to NSD-fed OZR and preserved by only SAC/C21 treatment (NSD 1.0 ± 0.15 vs HSD 0.39 ± 0.05 vs SAC/C21 0.82 ± 0.13) while ACE2 activity between the groups was unchanged (Figures 3G,H).

### RAS Components

**FIGURE 4:**
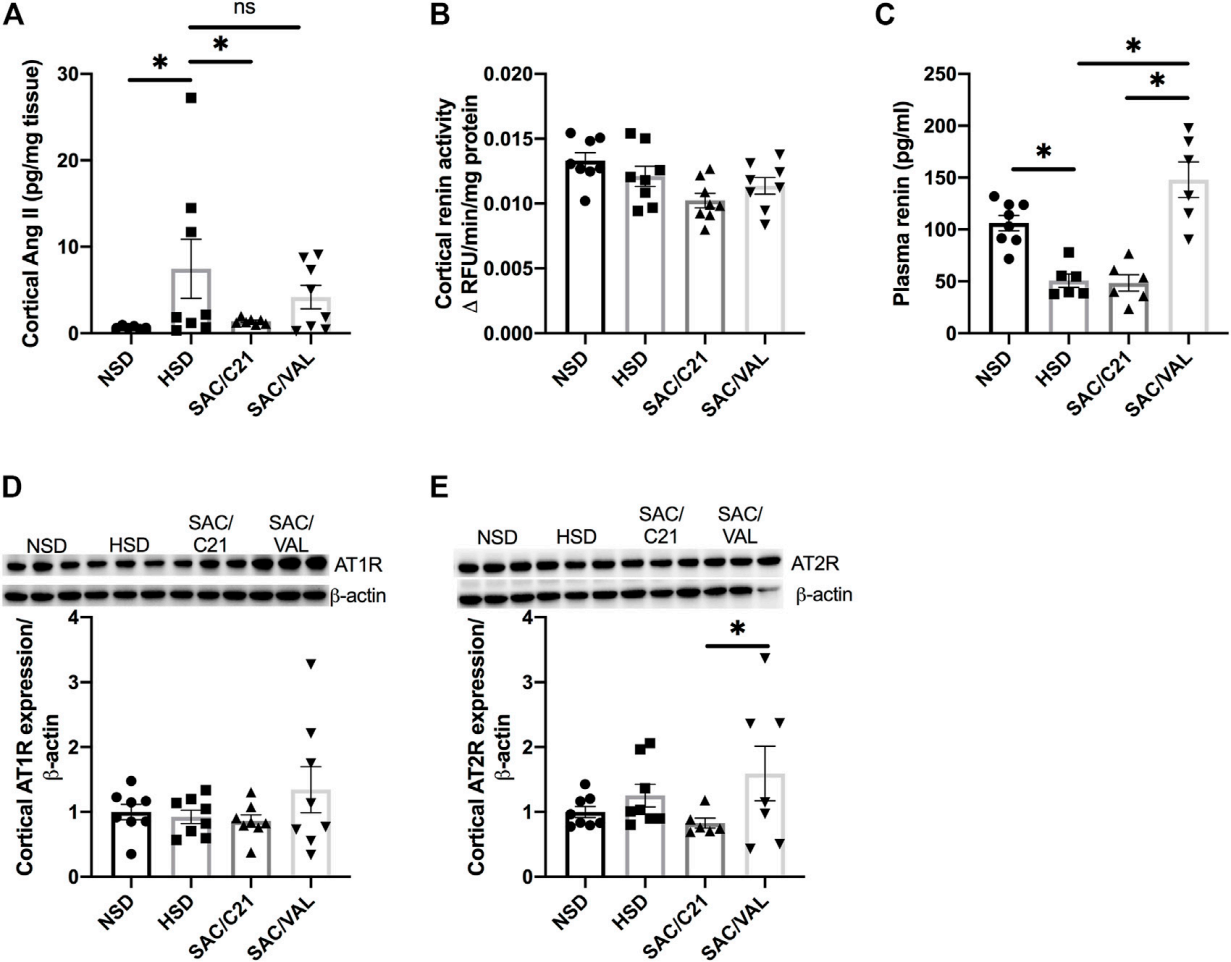
Angiotensin peptides, receptors and downstream signaling markers in obese Zucker rats on NSD or HSD and treated with vehicle, SAC/C21, or SAC/VAL. **(A)** cortical Ang II, **(B)** renin activity in kidney cortex, **(C)** plasma renin, **(D)** AT_1_R expression in kidney cortex, **(E)** AT_2_R expression in kidney cortex from obese Zucker rats on NSD or HSD and treated with vehicle, SAC/C21, or SAC/VAL. Data shown as mean ± SEM, analyzed by one-way ANOVA, followed by Fisher’s LSD test. Results are considered significant at *p < 0.05, with a 95% confidence interval; *n* = 6–8 for Ang II, plasma renin and AT_2_R expression and *n* = 8 renin activity and AT_1_R expression. NSD—normal salt diet fed + vehicle treated obese control, HSD—high salt diet fed + vehicle treated obese control, SAC/C21—high salt diet fed obese rat treated with sacubitril + C21, SAC/VAL—high salt diet fed obese rat treated with sacubitril + valsartan.

HSD-feeding caused a 5-fold increase in cortical levels of Ang II, which was significantly decreased by SAC/C21 but not SAC/VAL treatment (HSD 7.5 ± 3.4 vs SAC/C21 1.4 ± 0.1 and SAC/VAL 4.2 ± 1.3 pg/mg tissue) (Figure 4A). Only SAC/C21 treatment afforded a nearly significant reduction in cortical renin activity compared to HSD-fed OZR (HSD vs SAC/C21; *p* = 0.0538) (Figure 4B). Plasma renin was decreased by HSD feeding, but significantly increased by SAC/VAL treatment (HSD 50.7 ± 6.4 vs SAC/VAL 147.9 ± 17.1 pg/ml) (Figure 4C). There was a slight increase (not significant) in renal expression of AT_1_R in the kidney cortex of SAC/VAL treated rats, along with a significant increase in AT_2_R expression compared to SAC/C21 treated rats (Figures 4D,E).

## Discussion

This study highlights the superior renal benefits of a novel combinatory approach targeting neprilysin and AT_2_R, with the neprilysin inhibitor SAC and AT_2_R agonist C21 (SAC/C21), compared to SAC and AT_1_R blocker VAL (SAC/VAL) therapy in OZR. SAC/VAL therapy is commonly associated with albuminuria (Voors et al., 2015; Damman et al., 2018), a risk factor for and indicator of kidney injury. Obese rats develop kidney injury ∼9 weeks of age, evidenced by increases in urinary albumin excretion and mesangial matrix (Schmitz et al., 1992), and are a model known to be salt sensitive (Carlson et al., 2000; Pamidimukkala and Jandhyala, 2004). HSD-feeding accelerates the severity of injury to the kidney and onset of proteinuria shown by prior studies in our laboratory (Patel et al., 2016). We herein report for the first time that SAC/C21 prevented a decline in kidney function and afforded stronger reno-protection than SAC/VAL as evidenced by completely preventing proteinuria [measured with pyrogallol red-molybdate complex (Watanabe et al., 1986)] and albuminuria in HSD-fed OZR. Persistent proteinuria is a reliable risk factor for kidney failure that also plays a pathogenic role in the progression of kidney disease (Gorriz and Martinez-Castelao, 2012). On the other hand, albuminuria reflects an increased glomerular permeability (Benzing and Salant, 2021), which is suggestive of a greater degree of damage to the glomerulus that would occur during later stages of injury, and explains why urinary albumin is moderately though insignificantly increased in HSD-fed OZR compared to NSD-fed OZR. Nevertheless, the combination of SAC/C21 completely prevented or decreased albuminuria compared with HSD-fed animals.

Cystatin C, a protein that after being freely filtered at the glomerulus, is reabsorbed along the proximal tubules of the nephron and degraded. Thus, plasma cystatin C concentrations are inversely related to eGFR, and increased urinary concentrations are a reflection of tubular damage (Mijuskovic et al., 2007). Urinary cystatin C was increased by HSD, an effect prevented by both treatments, but by a greater degree of significance in SAC/C21 treated rats. On the other hand, the decreased plasma cystatin C concentrations in HSD-fed OZR are suggestive of hyperfiltration, a response corroborated by decreased plasma creatinine, increased urinary creatinine [based on high-performance liquid chromatography measurements (Yuen et al., 2004)] and eGFR in HSD-fed OZR. Taken together, 16 days of HSD-feeding in OZR was associated with renal hyperfiltration, a response that can be maladaptive over time in the progression of chronic kidney disease (CKD) (Brenner et al., 1996) and one that is typically associated with a progressive increase in albumin excretion rate (Palatini, 2012). The HSD-induced hyperfiltration was not prevented by either treatment, suggesting that the superior anti-proteinuric effects of SAC/C21 compared to SAC/VAL are independent of hyperfiltration. We acknowledge that measurements in this study were acquired after 16 days of HSD-feeding and treatment, and therefore do not know how SAC/C21 and SAC/VAL would affect these indices at later stages of CKD. As it relates to sex-difference, it cannot be definitively stated that these beneficial effects would be similar or greater in female OZR. Since AT_2_R is located on the X chromosome (Matavelli and Siragy, 2015) and has been shown to have greater kidney function in females (Hilliard et al., 2012; Hilliard et al., 2014), in part due to its higher renal expression (Armando et al., 2002), it’s likely that the benefit would be greater in females. However, for a definitive conclusion, a comparative study is warranted, and should also include older females with reduced estrogen levels.

Osteopontin is a glycoprotein normally expressed along the loop of Henle and distal convoluted tubules (Xie et al., 2001). Studies using rat models of accelerated anti-glomerular basement membrane glomerulonephritis (Lan et al., 1998), subtotal (5/6) renal mass ablation (Yu et al., 2000), and streptozotocin-induced diabetes (Fischer et al., 1998) have similarly concluded that with injury renal osteopontin expression is upregulated. The upregulated osteopontin in said models is correlated with proteinuria, and macrophage and monocyte accumulation within the kidney (Fischer et al., 1998; Lan et al., 1998; Yu et al., 2000) making its urinary excretion a biomarker of kidney injury (Phillips et al., 2016; Feldreich et al., 2017). Urinary osteopontin concentrations were increased by HSD-feeding, and among the HSD-fed groups were lowest in SAC/C21 treated OZR. To gain insight into the mechanism involved in the anti-proteinuric effects of SAC/C21, the expression of glomerular and tubular proteins was determined. Nephrin and podocin are proteins expressed by podocyte epithelial cells which are recognized for their critical role in the maintenance of the glomerular filtration barrier and slit diaphragm function (Levidiotis and Power, 2005). Interestingly, we found that renal nephrin expression was significantly increased upon HSD-feeding, an effect prevented by only SAC/C21 treatment. These changes occurred in parallel with proteinuria and albuminuria. Although the changes in renal expression of podocin and megalin, an endocytic receptor localized along the apical surface of proximal tubular epithelial cells for protein uptake (Mahadevappa et al., 2014) remained insignificant, the modest changes mirrored those of nephrin. Thus, it is likely that the increased fractional excretion of proteins led to a compensatory upregulation of nephrin (Schaefer et al., 2004), podocin, and megalin in HSD-fed and SAC/VAL groups, which was prevented by SAC/C21 treatment.

The renal AT_2_R and the reno-protective ACE2/Ang-(1–7) RAS axis have been increasingly studied in multiple animal models. ACE2 and neprilysin are peptidases that metabolize Ang II and Ang I, respectively into the reno-protective Ang-(1–7). However, neprilysin is also involved in one of the mechanisms of ANP elimination via enzymatic degradation; natriuretic peptide receptor type C (NPR-C) is involved in the receptor-mediated clearance of ANP (McMurray, 2015). Renal neprilysin activity was equally decreased in both treated groups and confirmed to be a direct effect of SAC because renal neprilysin expression was not changed by either treatment. However, as plasma ANP levels were unchanged by both treatments compared to HSD-fed OZR, NPR-C expression was measured in both the kidney and white adipose tissue (WAT), which in obesity is a major contributor to ANP clearance (Rubattu et al., 2010). The expression of NPR-C remained unchanged in the kidney and adipose tissue of all groups, suggesting NPR-C is not contributing to a greater ANP degradation in the treatment groups. Nevertheless, neprilysin inhibition via SAC in both of the treatment groups caused a modest increase in ANP compared with HSD-fed controls, which was not significantly different from NSD-fed OZR controls. It is worth it to mention that SAC treatment in combination with ACE inhibition was associated with high rates of angioedema (Kostis et al., 2004), resulting from increased bradykinin, a substrate of both neprilysin and ACE. Plasma bradykinin concentrations remained unchanged among the groups, suggesting that similar to SAC/VAL, SAC/C21 may also offer lower incidences of angioedema if pursued for further clinical investigation. As ACE2 is a known contributor to Ang-(1–7), ACE2 activity and expression were also measured. It was determined that while ACE2 expression was preserved in SAC/C21 treated OZR, the activity was only slightly, but non-significantly, increased. While almost all HSD-fed OZR controls had lower neprilysin expression than NSD-fed OZR, which was accompanied by a significant decrease in activity, this was not the case with ACE2 expression and activity. The explanation for the significant change in ACE2 expression but not activity could be the result of the phosphorylation status of ACE2. ACE2 activity has been reported to depend on the state of phosphorylation (Zhang et al., 2018), not on the expression alone, suggesting that the change in expression may not necessarily reflect enzyme activity.

Use of ARBs via a feedback loop drives a compensatory increase in plasma renin levels (Chen et al., 2010), in addition to increases in Ang I and Ang II (Gavras and Gavras, 1999). Azizi et al. have shown that combining VAL with the renin inhibitor aliskiren was essential to prevent increases in plasma Ang I, Ang II, and renin activity (Azizi et al., 2004). Therefore, the effects of SAC/C21 and SAC/VAL on the aforesaid RAS components along with the expression of angiotensin receptors were analyzed. Salt sensitivity in humans is commonly associated with low circulating renin levels (Richardson et al., 2013; Williams et al., 2014), which is in accordance with our data on plasma renin concentrations in salt sensitive HSD-fed OZR. On the other hand, circulating renin was significantly increased by only SAC/VAL treatment. Furthermore, while the expression of AT_1_Rs was moderately increased, the increase in AT_2_R expression was significant in SAC/VAL treated OZR compared to SAC/C21, which is perhaps related to treatment with VAL (Barker et al., 2006). Renal Ang II concentrations were not decreased by SAC/VAL treatment, suggesting the modest increase in AT_1_R expression was compensated for by a modest increase in AT_2_R expression, a consequence as mentioned that is likely to be associated with VAL therapy. Furthermore, cortical renin activity and total kidney weight were attenuated by only SAC/C21. The significance of these findings could be related to renin, and its precursor prorenin, which bind the (pro)renin receptor expressed in the kidney, heart, adipose, and other tissues of the body (Ames et al., 2019). Also, a decrease in kidney weight by SAC/C21 treatment could be explained in light of its comparison with the kidney weight of age matched lean Zucker rats (LZR). As obesity causes increases in kidney weight, OZR typically present with increased kidney weight compared with LZR (LZR 2.1 ± 0.15 vs OZR 2.6 ± 0.06 g). While HSD-feeding did not affect OZR weight, SAC/C21 treatment did attenuate increases in kidney weight, which may have physiological relevance. However, to address this important question a detailed study evaluating changes in kidney structure is needed. While the exact involvement in the pathophysiological mechanisms of renal and/or cardiovascular disease are not yet fully understood, it is known that high renin levels also have detrimental effects independent from Ang II, via binding (pro)renin receptor (Nguyen et al., 2004). Moreover, high concentrations of tissue Ang II induce pro-fibrotic, pro-inflammatory, and pro-hypertrophic effects that damage the kidney (Ames et al., 2019), influence urinary excretion of albumin (Clavant et al., 2003), and increased the fractional excretion of protein (Lapinski et al., 1996). Despite the involvement of alternate pathways involving enzymes such as chymase in the generation of Ang II, the predominant enzymes involved in Ang II generation include renin and ACE. While ARBs like VAL inhibit the deleterious actions of Ang II at AT_1_R regardless of its origin, studies have found that use of ARBs is associated with significant increases in Ang II, and that when given in combination with ACE inhibitors, ACE inhibition dose-dependently prevents the increases in Ang II induced by AT_1_R blockade (Ménard et al., 1997). Other reports have found differences in the ability of ARBs to produce sustained vascular and intrarenal blockade of AT_1_Rs (Coltamai et al., 2010). This evidence, taken together with the increased renal Ang II levels and increased renal AT_1_R expression in SAC/VAL treated OZR suggests that it is likely that the dose of VAL used in this study might not have been sufficient to offset and block the AT_1_R from the 3-fold increase in intrarenal Ang II levels. Though this should be taken into consideration for the development of reno-protective approaches, our study presents an innovative approach that eliminates the kidney specific problems associated with ARB monotherapy and SAC/VAL therapy via a novel combination of SAC with C21.

## Data Availability

The original contributions presented in the study are included in the article/Supplementary Material, further inquiries can be directed to the corresponding author.

## References

[B1] Ali Q. Hussain T. (2012). AT2 Receptor Non-peptide Agonist C21 Promotes Natriuresis in Obese Zucker Rats. Hypertens. Res. 35, 654–660. 10.1038/hr.2012.13 22297475 PMC3912844

[B2] Ali Q. Patel S. Hussain T. (2015). Angiotensin AT2 Receptor Agonist Prevents Salt-Sensitive Hypertension in Obese Zucker Rats. Am. J. Physiol. Ren. Physiol. 308, F1379–F1385. 10.1152/ajprenal.00002.2015 PMC4469886 25855512

[B3] Ames M. K. Atkins C. E. Pitt B. (2019). The Renin-Angiotensin-Aldosterone System and its Suppression. J. Vet. Intern. Med. 33, 363–382. 10.1111/jvim.15454 30806496 PMC6430926

[B4] Armando I. Jezova M. Juorio A. V. Terrón J. A. Falcón-Neri A. Semino-Mora C. (2002). Estrogen Upregulates Renal Angiotensin II AT(2) Receptors. Am. J. Physiol. Ren. Physiol 283, F934–F943. 10.1152/ajprenal.00145.2002 12372768

[B5] Azizi M. Ménard J. Bissery A. Guyenne T. T. Bura-Rivière A. Vaidyanathan S. (2004). Pharmacologic Demonstration of the Synergistic Effects of a Combination of the Renin Inhibitor Aliskiren and the AT1 Receptor Antagonist Valsartan on the Angiotensin II-Renin Feedback Interruption. J. Am. Soc. Nephrol. 15, 3126–3133. 10.1097/01.ASN.0000146686.35541.29 15579516

[B6] Barker T. A. Massett M. P. Korshunov V. A. Mohan A. M. Kennedy A. J. Berk B. C. (2006). Angiotensin II Type 2 Receptor Expression after Vascular Injury: Differing Effects of Angiotensin-Converting Enzyme Inhibition and Angiotensin Receptor Blockade. Hypertension 48, 942–949. 10.1161/01.HYP.0000241061.51003.b7 16982965

[B7] Benzing T. Salant D. (2021). Insights into Glomerular Filtration and Albuminuria. N. Engl. J. Med. 384, 1437–1446. 10.1056/NEJMra1808786 33852781

[B8] Brenner B. M. Lawler E. V. Mackenzie H. S. (1996). The Hyperfiltration Theory: a Paradigm Shift in Nephrology. Kidney Int. 49, 1774–1777. 10.1038/ki.1996.265 8743495

[B9] Carlson S. H. Shelton J. White C. R. Wyss J. M. (2000). Elevated Sympathetic Activity Contributes to Hypertension and Salt Sensitivity in Diabetic Obese Zucker Rats. Hypertension 35, 403–408. 10.1161/01.hyp.35.1.403 10642332

[B10] Chen L. Kim S. M. Eisner C. Oppermann M. Huang Y. Mizel D. (2010). Stimulation of Renin Secretion by Angiotensin II Blockade Is Gsalpha-dependent. J. Am. Soc. Nephrol. 21, 986–992. 10.1681/ASN.2009030307 20395378 PMC2900955

[B11] Clavant S. P. Forbes J. M. Thallas V. Osicka T. M. Jerums G. Comper W. D. (2003). Reversible Angiotensin II-Mediated Albuminuria in Rat Kidneys Is Dynamically Associated with Cytoskeletal Organization. Nephron Physiol. 93, p51–60. 10.1159/000068528 12629271

[B12] Coltamai L. Maillard M. Simon A. Vogt B. Burnier M. (2010). Comparative Vascular and Renal Tubular Effects of Angiotensin II Receptor Blockers Combined with a Thiazide Diuretic in Humans. J. Hypertens. 28, 520–526. 10.1097/HJH.0b013e3283346be1 20104189

[B13] Damman K. Gori M. Claggett B. Jhund P. S. Senni M. Lefkowitz M. P. (2018). Renal Effects and Associated Outcomes during Angiotensin-Neprilysin Inhibition in Heart Failure. JACC Heart Fail. 6, 489–498. 10.1016/j.jchf.2018.02.004 29655829

[B14] Feldreich T. Carlsson A. C. Helmersson-Karlqvist J. Risérus U. Larsson A. Lind L. (2017). Urinary Osteopontin Predicts Incident Chronic Kidney Disease, while Plasma Osteopontin Predicts Cardiovascular Death in Elderly Men. Cardiorenal Med. 7, 245–254. 10.1159/000476001 28736565 PMC5511996

[B15] Fischer J. W. Tschöpe C. Reinecke A. Giachelli C. M. Unger T. (1998). Upregulation of Osteopontin Expression in Renal Cortex of Streptozotocin-Induced Diabetic Rats Is Mediated by Bradykinin. Diabetes 47, 1512–1518. 10.2337/diabetes.47.9.1512 9726243

[B16] Gavras I. Gavras H. (1999). Effects of Eprosartan versus Enalapril in Hypertensive Patients on the Renin-Angiotensin-Aldosterone System and Safety Parameters: Results from a 26-week, Double-Blind, Multicentre Study. Eprosartan Multinational Study Group. Curr. Med. Res. Opin. 15, 15–24. 10.1185/03007999909115169 10216807

[B17] George P. M. Wells A. U. Jenkins R. G. (2020). Pulmonary Fibrosis and COVID-19: the Potential Role for Antifibrotic Therapy. Lancet Respir. Med. 8, 807–815. 10.1016/S2213-2600(20)30225-3 32422178 PMC7228727

[B18] Gorriz J. L. Martinez-Castelao A. (2012). Proteinuria: Detection and Role in Native Renal Disease Progression. Transpl. Rev (Orlando). 26, 3–13. 10.1016/j.trre.2011.10.002 22137726

[B19] Hall J. E. Mouton A. J. Da Silva A. A. Omoto A. Wang Z. Li X. (2020). Obesity, Kidney Dysfunction, and Inflammation: Interactions in Hypertension. Cardiovasc. Res. 117, 1859–1876. 10.1093/cvr/cvaa336 PMC8262632 33258945

[B20] Haynes R. Judge P. K. Staplin N. Herrington W. G. Storey B. C. Bethel A. (2018). Effects of Sacubitril/Valsartan versus Irbesartan in Patients with Chronic Kidney Disease: A Randomized Double-Blind Trial. Circulation. 138, 1505–1514. 10.1161/CIRCULATIONAHA.118.034818 30002098

[B21] Helin K. Tikkanen I. Tikkanen T. Saijonmaa O. Sybertz E. J. Vemulapalli S. (1991). Prolonged Neutral Endopeptidase Inhibition in Heart Failure. Eur. J. Pharmacol. 198, 23–30. 10.1016/0014-2999(91)90557-7 1655477

[B22] Helmer A. Slater N. Smithgall S. (2018). A Review of ACE Inhibitors and ARBs in Black Patients with Hypertension. Ann. Pharmacother. 52, 1143–1151. 10.1177/1060028018779082 29808707

[B23] Hilliard L. M. Chow C. L. Mirabito K. M. Steckelings U. M. Unger T. Widdop R. E. (2014). Angiotensin Type 2 Receptor Stimulation Increases Renal Function in Female, but Not Male, Spontaneously Hypertensive Rats. Hypertension. 64, 378–383. 10.1161/HYPERTENSIONAHA.113.02809 24842923

[B24] Hilliard L. M. Jones E. S. Steckelings U. M. Unger T. Widdop R. E. Denton K. M. (2012). Sex-specific Influence of Angiotensin Type 2 Receptor Stimulation on Renal Function: a Novel Therapeutic Target for Hypertension. Hypertension. 59, 409–414. 10.1161/HYPERTENSIONAHA.111.184986 22158645

[B25] Kostis J. B. Packer M. Black H. R. Schmieder R. Henry D. Levy E. (2004). Omapatrilat and Enalapril in Patients with Hypertension: the Omapatrilat Cardiovascular Treatment vs. Enalapril (OCTAVE) Trial. Am. J. Hypertens. 17, 103–111. 10.1016/j.amjhyper.2003.09.014 14751650

[B26] Koulis C. Chow B. S. Mckelvey M. Steckelings U. M. Unger T. Thallas-Bonke V. (2015). AT2R Agonist, Compound 21, Is reno-protective against Type 1 Diabetic Nephropathy. Hypertension 65, 1073–1081. 10.1161/HYPERTENSIONAHA.115.05204 25776077

[B27] Kusaka H. Sueta D. Koibuchi N. Hasegawa Y. Nakagawa T. Lin B. (2015). LCZ696, Angiotensin II Receptor-Neprilysin Inhibitor, Ameliorates High-Salt-Induced Hypertension and Cardiovascular Injury More Than Valsartan Alone. Am. J. Hypertens. 28, 1409–1417. 10.1093/ajh/hpv015 25762811

[B28] Lan H. Y. Yu X. Q. Yang N. Nikolic-Paterson D. J. Mu W. Pichler R. (1998). De Novo glomerular Osteopontin Expression in Rat Crescentic Glomerulonephritis. Kidney Int. 53, 136–145. 10.1046/j.1523-1755.1998.00748.x 9453010

[B29] Lapinski R. Perico N. Remuzzi A. Sangalli F. Benigni A. Remuzzi G. (1996). Angiotensin II Modulates Glomerular Capillary Permselectivity in Rat Isolated Perfused Kidney. J. Am. Soc. Nephrol. 7, 653–660. 10.1681/ASN.V75653 8738798

[B30] Levidiotis V. Power D. A. (2005). New Insights into the Molecular Biology of the Glomerular Filtration Barrier and Associated Disease. Nephrology (Carlton). 10, 157–166. 10.1111/j.1440-1797.2005.00385.x 15877676

[B31] Mahadevappa R. Nielsen R. Christensen E. I. Birn H. (2014). Megalin in Acute Kidney Injury: Foe and Friend. Am. J. Physiol. Ren. Physiol 306, F147–F154. 10.1152/ajprenal.00378.2013 24197071

[B32] Maric-Bilkan C. (2013). Obesity and Diabetic Kidney Disease. Med. Clin. North. Am. 97, 59–74. 10.1016/j.mcna.2012.10.010 23290730 PMC3539140

[B33] Matavelli L. C. Siragy H. M. (2015). AT2 Receptor Activities and Pathophysiological Implications. J. Cardiovasc. Pharmacol. 65, 226–232. 10.1097/FJC.0000000000000208 25636068 PMC4355033

[B34] Mcmurray J. J. (2015). Neprilysin Inhibition to Treat Heart Failure: a Tale of Science, Serendipity, and Second Chances. Eur. J. Heart Fail. 17, 242–247. 10.1002/ejhf.250 25756942

[B35] Ménard J. Campbell D. J. Azizi M. Gonzales M. F. (1997). Synergistic Effects of ACE Inhibition and Ang II Antagonism on Blood Pressure, Cardiac Weight, and Renin in Spontaneously Hypertensive Rats. Circulation. 96, 3072–3078. 10.1161/01.cir.96.9.3072 9386177

[B36] Mijušković Z. Maksić Đ. Hrvačević R. Vučelić M. Subota V. Stojanović J. (2007). Urinary Cystatin C as a Marker of Tubular Dysfunction. J. Med. Biochem. 26, 98–102. 10.2478/v10011-007-0013-9

[B37] Nguyen G. Burcklé C. A. Sraer J. D. (2004). Renin/prorenin-receptor Biochemistry and Functional Significance. Curr. Hypertens. Rep. 6, 129–132. 10.1007/s11906-004-0088-3 15010017

[B38] Palatini P. (2012). Glomerular Hyperfiltration: a Marker of Early Renal Damage in Pre-diabetes and Pre-hypertension. Nephrol. Dial. Transplant. 27, 1708–1714. Oxford University Press. 10.1093/ndt/gfs037 22431709

[B39] Pamidimukkala J. Jandhyala B. S. (2004). Effects of Salt Rich Diet in the Obese Zucker Rats: Studies on Renal Function during Isotonic Volume Expansion. Clin. Exp. Hypertens. 26, 55–67. 10.1081/ceh-120027331 15000297

[B40] Pandey A. Gaikwad A. B. (2017). AT2 Receptor Agonist Compound 21: a Silver Lining for Diabetic Nephropathy. Eur. J. Pharmacol. 815, 251–257. 10.1016/j.ejphar.2017.09.036 28943106

[B41] Patel S. N. Ali Q. Hussain T. (2016). Angiotensin II Type 2-Receptor Agonist C21 Reduces Proteinuria and Oxidative Stress in Kidney of High-Salt-Fed Obese Zucker Rats. Hypertension. 67, 906–915. 10.1161/HYPERTENSIONAHA.115.06881 27021008 PMC4833537

[B42] Phillips J. A. Holder D. J. Ennulat D. Gautier J. C. Sauer J. M. Yang Y. (2016). Rat Urinary Osteopontin and Neutrophil Gelatinase-Associated Lipocalin Improve Certainty of Detecting Drug-Induced Kidney Injury. Toxicol. Sci. 151, 214–223. 10.1093/toxsci/kfw038 27026710

[B43] Richards A. M. Wittert G. A. Crozier I. G. Espiner E. A. Yandle T. G. Ikram H. (1993). Chronic Inhibition of Endopeptidase 24.11 in Essential Hypertension: Evidence for Enhanced Atrial Natriuretic Peptide and Angiotensin II. J. Hypertens. 11, 407–416. 10.1097/00004872-199304000-00011 8390508

[B44] Richardson S. I. Freedman B. I. Ellison D. H. Rodriguez C. J. (2013). Salt Sensitivity: a Review with a Focus on Non-hispanic Blacks and Hispanics. J. Am. Soc. Hypertens. 7, 170–179. 10.1016/j.jash.2013.01.003 23428408 PMC4574876

[B45] Rubattu S. Sciarretta S. Morriello A. Calvieri C. Battistoni A. Volpe M. (2010). NPR-C: a Component of the Natriuretic Peptide Family with Implications in Human Diseases. J. Mol. Med. (Berl) 88, 889–897. 10.1007/s00109-010-0641-2 20563546

[B46] Rutkowski P. Klassen A. Sebekova K. Bahner U. Heidland A. (2006). Renal Disease in Obesity: the Need for Greater Attention. J. Ren. Nutr. 16, 216–223. 10.1053/j.jrn.2006.04.017 16825023

[B47] Schaefer L. Ren S. Schaefer R. M. Mihalik D. Babelova A. Huwiler A. (2004). Nephrin Expression Is Increased in anti-Thy1.1-induced Glomerulonephritis in Rats. Biochem. Biophys. Res. Commun. 324, 247–254. 10.1016/j.bbrc.2004.09.042 15465010

[B48] Schiffrin E. L. Lipman M. L. Mann J. F. (2007). Chronic Kidney Disease: Effects on the Cardiovascular System. Circulation 116, 85–97. 10.1161/CIRCULATIONAHA.106.678342 17606856

[B49] Schmitz P. G. O'Donnell M. P. Kasiske B. L. Katz S. A. Keane W. F. (1992). Renal Injury in Obese Zucker Rats: Glomerular Hemodynamic Alterations and Effects of Enalapril. Am. J. Physiol. 263, F496–F502. 10.1152/ajprenal.1992.263.3.F496 1415578

[B50] Shimosawa T. (2013). Salt, the Renin-Angiotensin-Aldosterone System and Resistant Hypertension. Hypertens. Res. 36, 657–660. 10.1038/hr.2013.69 23912973

[B51] Tikellis C. Pickering R. Tsorotes D. du X. J. Kiriazis H. Nguyen-Huu T. P. (2012). Interaction of Diabetes and ACE2 in the Pathogenesis of Cardiovascular Disease in Experimental Diabetes. Clin. Sci. (Lond). 123, 519–529. 10.1042/CS20110668 22616805

[B52] Voors A. A. Gori M. Liu L. C. Claggett B. Zile M. R. Pieske B. (2015). Renal Effects of the Angiotensin Receptor Neprilysin Inhibitor LCZ696 in Patients with Heart Failure and Preserved Ejection Fraction. Eur. J. Heart Fail. 17, 510–517. 10.1002/ejhf.232 25657064

[B53] Watanabe N. Kamei S. Ohkubo A. Yamanaka M. Ohsawa S. Makino K. (1986). Urinary Protein as Measured with a Pyrogallol Red-Molybdate Complex, Manually and in a Hitachi 726 Automated Analyzer. Clin. Chem. 32, 1551–1554. 10.1093/clinchem/32.8.1551 3731450

[B54] Wenzel R. R. (2005). Renal protection in Hypertensive Patients: Selection of Antihypertensive Therapy. Drugs. 65 (Suppl. 2), 29–39. 10.2165/00003495-200565002-00005 16398060

[B55] Williams S. F. Nicholas S. B. Vaziri N. D. Norris K. C. (2014). African Americans, Hypertension and the Renin Angiotensin System. World J. Cardiol. 6, 878–889. 10.4330/wjc.v6.i9.878 25276290 PMC4176798

[B56] Xie Y. Sakatsume M. Nishi S. Narita I. Arakawa M. Gejyo F. (2001). Expression, Roles, Receptors, and Regulation of Osteopontin in the Kidney. Kidney Int. 60, 1645–1657. 10.1046/j.1523-1755.2001.00032.x 11703581

[B57] Yim H. E. Yoo K. H. (2008). Renin-Angiotensin System - Considerations for Hypertension and Kidney. Electrolyte Blood Press. 6, 42–50. 10.5049/EBP.2008.6.1.42 24459521 PMC3894487

[B58] Yu X. Q. Wu L. L. Huang X. R. Yang N. Gilbert R. E. Cooper M. E. (2000). Osteopontin Expression in Progressive Renal Injury in Remnant Kidney: Role of Angiotensin II. Kidney Int. 58, 1469–1480. 10.1046/j.1523-1755.2000.00309.x 11012882

[B59] Yuen P. S. Dunn S. R. Miyaji T. Yasuda H. Sharma K. Star R. A. (2004). A Simplified Method for HPLC Determination of Creatinine in Mouse Serum. Am. J. Physiol. Ren. Physiol. 286, F1116–F1119. 10.1152/ajprenal.00366.2003 14970000

[B60] Zhang H. Han G. W. Batyuk A. Ishchenko A. White K. L. Patel N. (2017). Structural Basis for Selectivity and Diversity in Angiotensin II Receptors. Nature. 544, 327–332. 10.1038/nature22035 28379944 PMC5525545

[B61] Zhang J. Dong J. Martin M. He M. Gongol B. Marin T. L. (2018). AMP-activated Protein Kinase Phosphorylation of Angiotensin-Converting Enzyme 2 in Endothelium Mitigates Pulmonary Hypertension. Am. J. Respir. Crit. Care Med. 198, 509–520. 10.1164/rccm.201712-2570OC 29570986 PMC6118028

