# Supplementary material for: Combining Neprilysin Inhibitor With AT2R Agonist Is Superior to Combination With AT1R Blocker in Providing Reno-Protection in Obese Rats
Source: Front Pharmacol. 2022 Feb 7;12:778953. doi: 10.3389/fphar.2021.778953 (PMC8859315; doi:10.3389/fphar.2021.778953)
Supplement: Supplementary file 1 [file DataSheet1.docx]

**Supplementary Material**

**Combining Neprilysin Inhibitor With AT_2_R Agonist Is Superior to Combination With AT_1_R Blocker in Providing Reno-Protection in Obese Rats**

Elizabeth Alana Gray^1^, Sanket N. Patel^1^, Peter A. Doris^2^ and Tahir Hussain^1*^

^1^Department of Pharmacological and Pharmaceutical Sciences, College of Pharmacy, University of Houston, Houston, TX 77204.

^2^The Brown Foundation Institute of Molecular Medicine Center for Human Genetics, The University of Texas Health Science Center at Houston, Houston TX 77030.

Short Title: Reno-protection by SAC/C21 in OZR

*Address for correspondence: Tahir Hussain, Ph.D., Associate Dean of Research and Graduate Programs, Professor of Pharmacology, 4849 Calhoun Rd. Health Building 2, Room 3044, University of Houston, Houston, TX 77204; [thussain@central.uh.edu](mailto:thussain@central.uh.edu); Phone 713-743-1276

*Indices of kidney function and injury:*

Proteinuria was measured using the Pyrogallol Red-Molybdate complex method.^1^ Briefly, all solutions were prepared and pH was adjusted to 2.5 using HCl. A linear curve was generated using bovine serum albumin standards (0.25 mg/ml to 4 mg/ml). Five μl of standards, blank and urine samples were added to wells in triplicates, followed by 300μl of pyrogallol red-molybdate solution. Absorbance was read at 600nm and protein concentrations normalized to 24 hour urine volume. Urinary samples were diluted 1:32 for measuring albumin with the Nephrat II Rat albumin ELISA kit (NR002, Ethos Biosciences), and the data similarly normalized to 24 hour urine volume. Urine creatinine was measured by HPLC following the method established by Yuen *et al*.^2^ Briefly, to prepare, all urine samples were initially diluted 1:5, and then further diluted into 10mM sodium acetate buffer (pH 5.1) for analysis. DetectX Serum Creatinine Kit (KB02-H1) was used to measure plasma creatinine. Plasma sodium concentrations were determined via atomic absorption spectroscopy with a Perkin Elmer S10 Autosampler and AAnalyst 400. Standards (0.05 mg/L – 2 mg/L) were generated using Perkin Elmer Pure Sodium (Na[7440-23-5]). Estimated glomerular filtration rate (eGFR) in ml/min was estimated using the following formula: (urine volume * urine creatinine)/plasma creatinine.

*Enzyme-linked immunosorbent assays:*

RayBiotech (EIA-ANP-1) ANP competition based ELISA was used to quantify ANP in plasma samples. Renin concentrations in plasma were determined using the RayBiotech ELR-Renin1-1 quantitative sandwich-based ELISA, as per manufacturers protocol. Mouse/Rat Osteopontin Quantikine ELISA kit (MOST00 R&D Systems) was used to determine concentrations of OPN in urine samples following the manufacturers protocol, data was then normalized to 24 hour urine volume. Mouse/Rat Cystatin C Immunoassay Quantikine ELISA (MSCTC0) was used for quantification of urinary and plasma cystatin C concentrations. Plasma bradykinin concentrations were acquired using the rat bradykinin ELISA colorimetric kit (NBP2-69971) from Novus Biologicals. Ang II was measured using BMA Biomedicals ELISA kit (S-1133). For Ang II ELISA, kidney cortices were homogenized in TRIS buffer, containing protease and phosphatase inhibitors (Pierce A32959), peptides were extracted through solid phase extraction (kit from BMA Biomedicals, S-5000), and then samples were vacuumed dry using a centrifugal vacufuge to be stored at -80° C until analysis. All samples were reconstituted in ELISA buffer for the assay.

*Enzyme Activities:*

SensoLyte 520 Neprilysin Activity assay kit (AS-72223) was used to determine neprilysin activity in kidney homogenates. Renin activity was similarly measured using the SensoLyte 520 Rat Renin assay kit (AS-72140) and ACE2 activity with SensoLyte 390 ACE2 Activity Assay kit (AS-72086). Kidney cortices were homogenized using the assay buffer provided in the kits, to which 0.05% Triton-X 100 was added. All samples and reagents once loaded into the plate were pre-incubated for 10 minutes and subsequently maintained at 37° C during fluorescent intensity measurements collected every 5 minutes at excitation and emission wavelengths of 490nm and 520nm.

*Western Blots:*

Kidney cortices and epididymal white adipose tissue were homogenized in radioimmunoprecipitation assay (RIPA) buffer, centrifuged at 10,000RPM for 10 minutes at 4º Celsius and supernatant collected from the samples. For the determination of megalin expression in kidney cortices, tissues were homogenized in RIPA buffer in which the amount of NP-40, Triton X-100 and SDS were all increased to 1%. For all kidney samples, 30μg of protein was loaded, 40μg were loaded from adipose samples. All kidney samples were loaded into Bio-Rad Mini-Protean TGX 4-20% gradient gels, except for samples used to detect megalin and podocin, which were loaded into NuPAGE 3-8% Tris-Acetate Gels (Invitrogen). Adipose samples were loaded into Bio-Rad 10% gels. All were transferred onto PVDF membranes (Bio-Rad 1620177) and developed with Bio-Rad Clarity Western ECL substrate in Bio-Rad ChemiDoc MP imaging system. The primary antibodies used included: Megalin (sc-515750) 1:1000; Nephrin (ab216341) 1:1000; Podocin (P0372-200ul) 1:1000; Neprilysin (ab261729) 1:1000; ACE2 (ab108252) 1:1000; NPR-C (ab177954) 1:1000; AT1R (ab124734) 1:1000; AT2R (ab92445) 1:1000; Beta-actin (ab8226) 1:4000; Beta-tubulin (protein tech 66240-I-Ig) 1:1000. Concentrations of secondary antibodies from Invitrogen goat anti-rabbit (31460) and goat anti-mouse (31430) used were 1:2000 for expression of all proteins except for beta-actin for which 1:4000 was used.

**A B C**
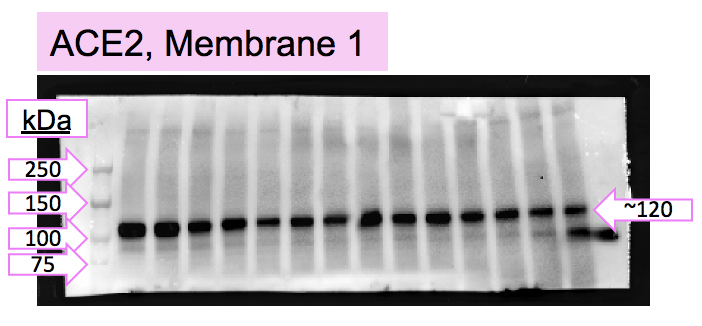

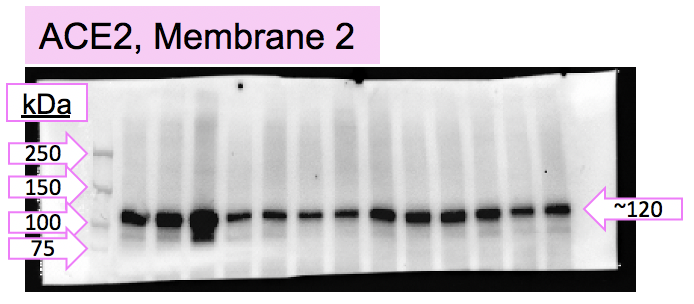

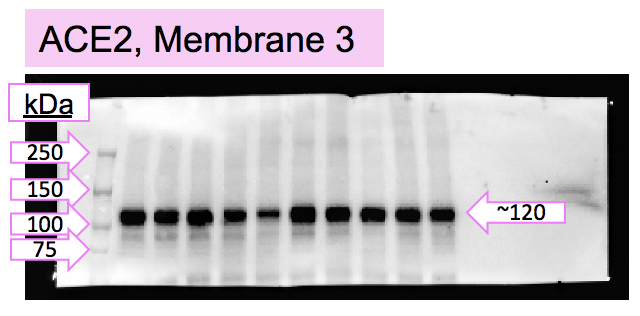


**D E F**
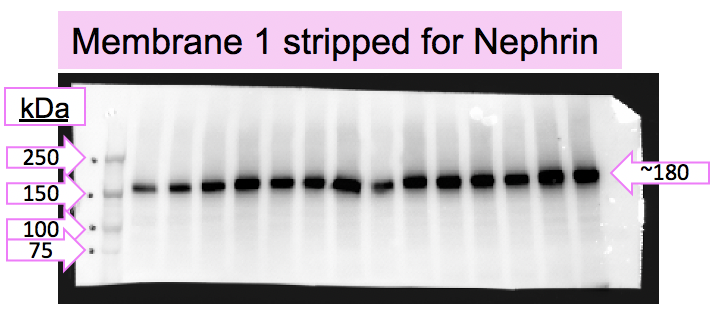

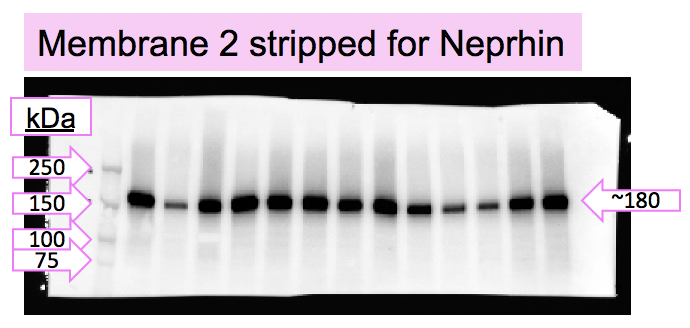

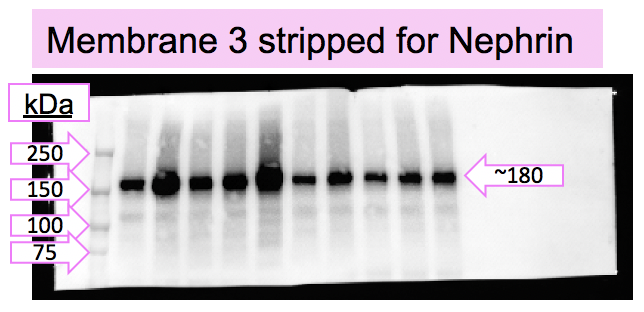


**G H I**


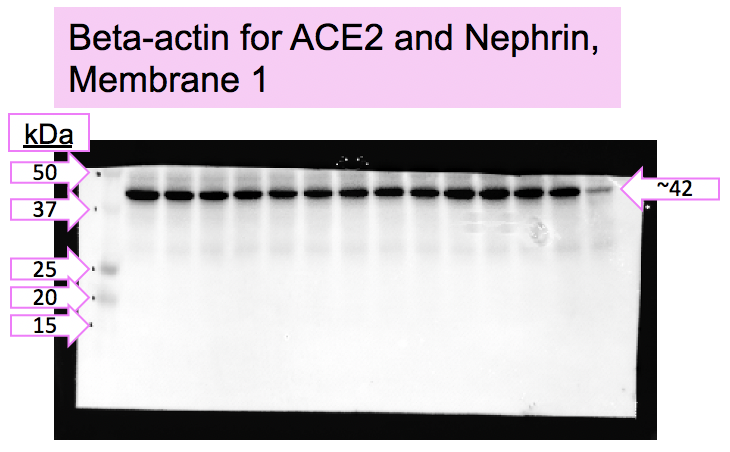

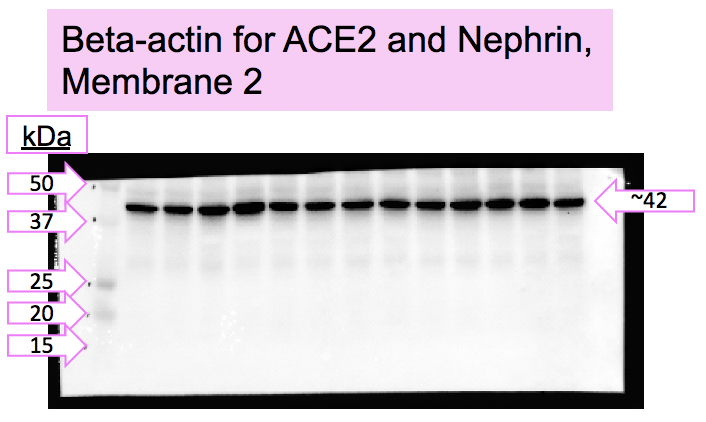

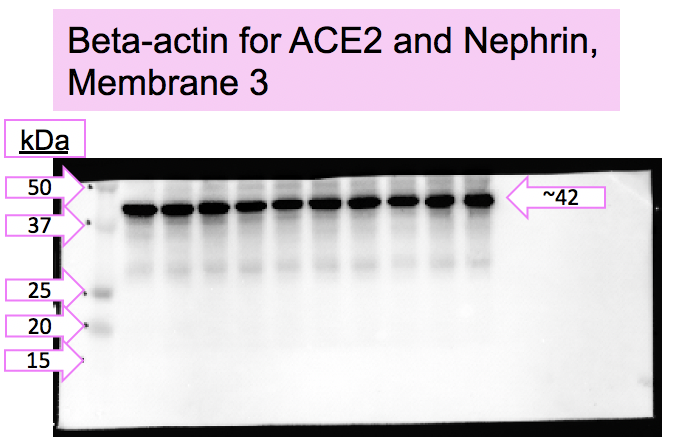


Supplementary Figure 1: Western blot membranes of renal ACE2 (A, B, & C), renal nephrin (D, E, & F), and loading control beta-actin (G, H, & I). Protein ladder was loaded into lane 1 of all three membranes, NSD loaded into lanes 3, 4 & 5 of membranes 1 & 2, and lanes 4 & 5 of membrane 3, HSD loaded into lanes 6, 7, & 8 of membranes 1 & 2, and lanes 6 & 7 of membrane 3, SAC/C21 into lanes 9, 10, & 11 of membranes 1 & 2, and lanes 8 & 9 of membrane 3, SAC/VAL into lanes 12, 13, & 14 of membranes 1 & 2, and lanes 10 & 11 of membrane 3.

**A B C**


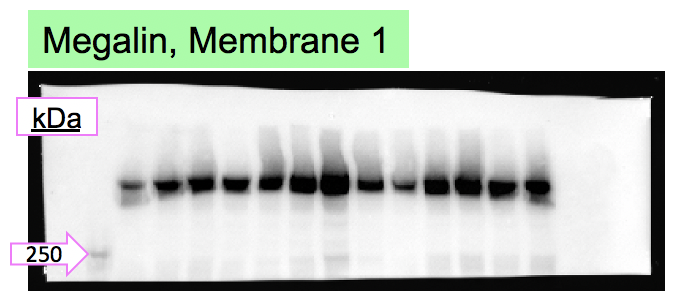

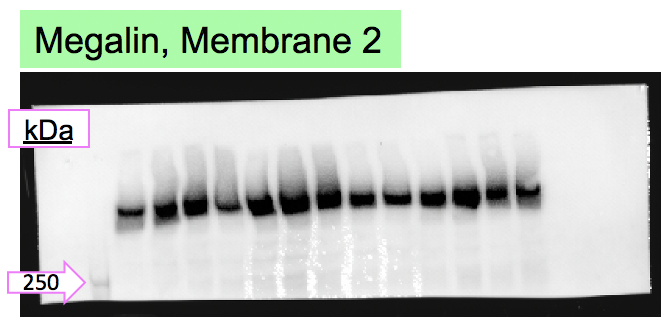

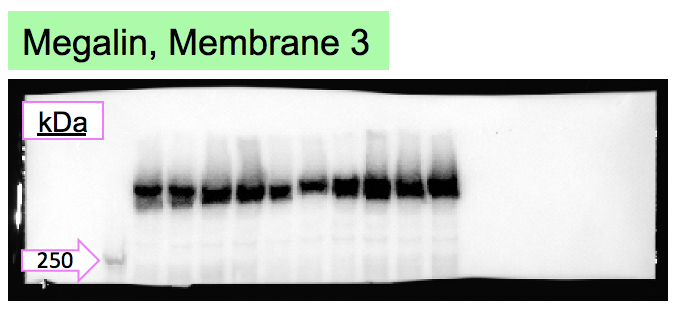


**D E F**


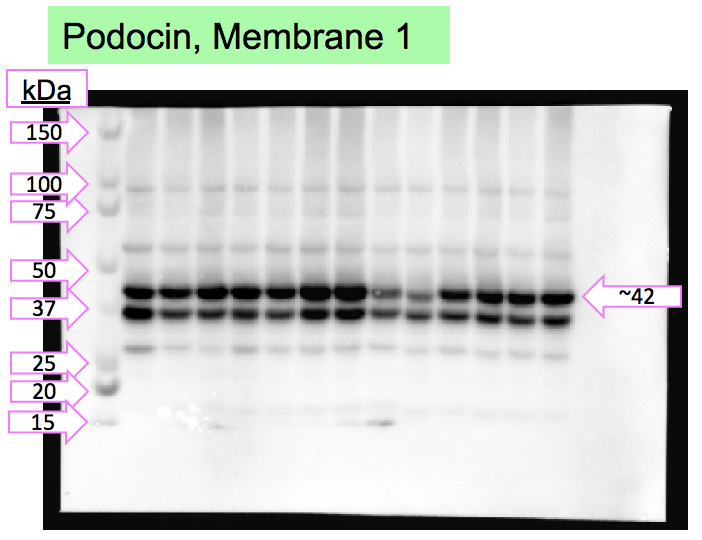

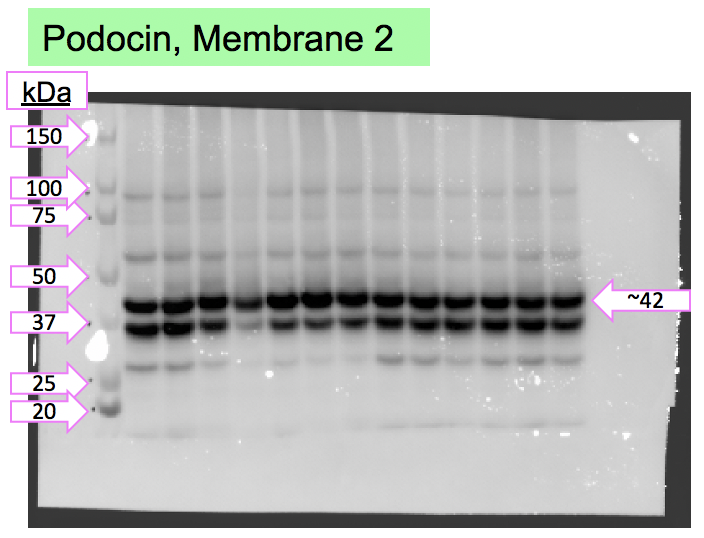

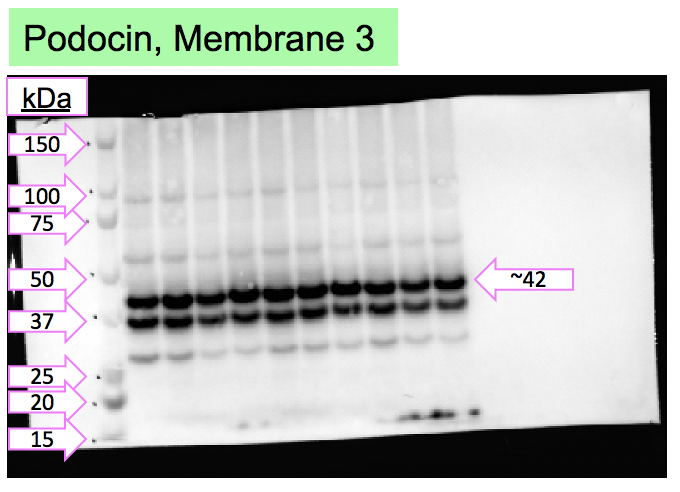


**G H I**


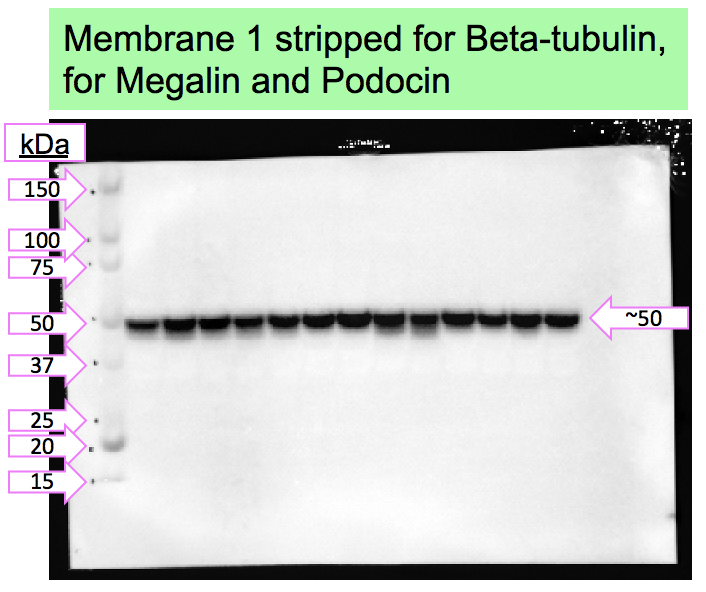

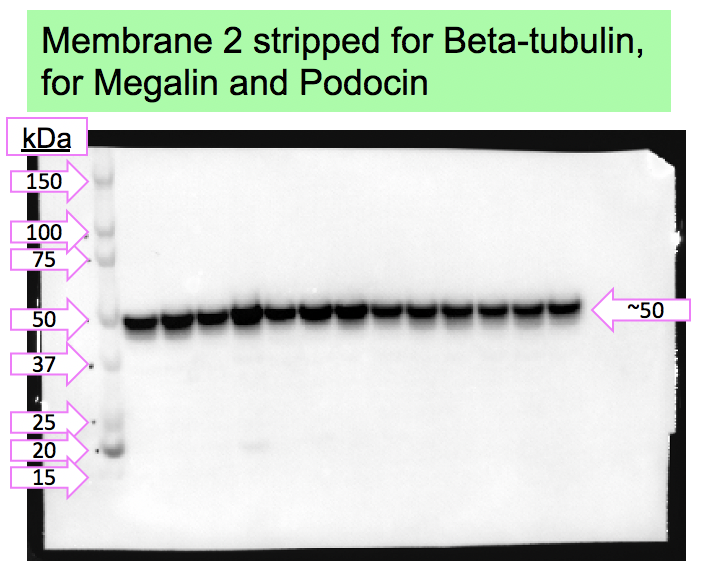

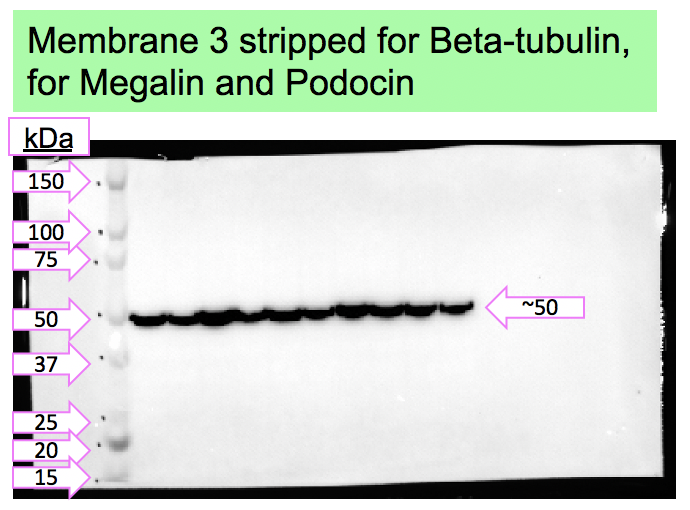


Supplementary Figure 2: Western blot membranes of renal megalin (A, B & C), renal podocin (D, E, & F), and loading control beta-tubulin (G, H, & I). Protein ladder was loaded into lane 1 of all three membranes, NSD loaded into lanes 3, 4 & 5 of membranes 1 & 2, and lanes 4 & 5 of membrane 3, HSD loaded into lanes 6, 7, & 8 of membranes 1 & 2, and lanes 6 & 7 of membrane 3, SAC/C21 into lanes 9, 10, & 11 of membranes 1 & 2, and lanes 8 & 9 of membrane 3, SAC/VAL into lanes 12, 13, & 14 of membranes 1 & 2, and lanes 10 & 11 of membrane 3.

**A B C**


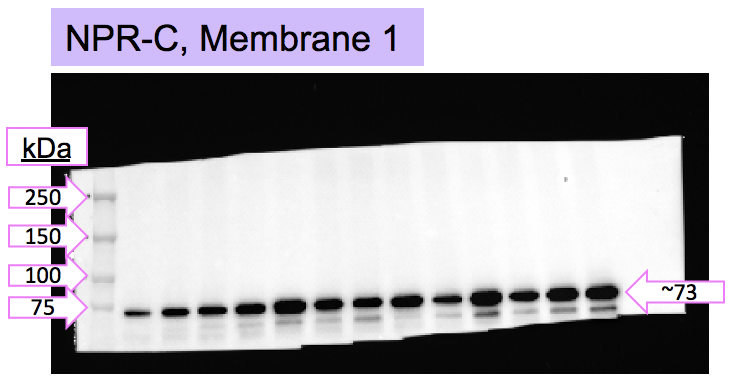

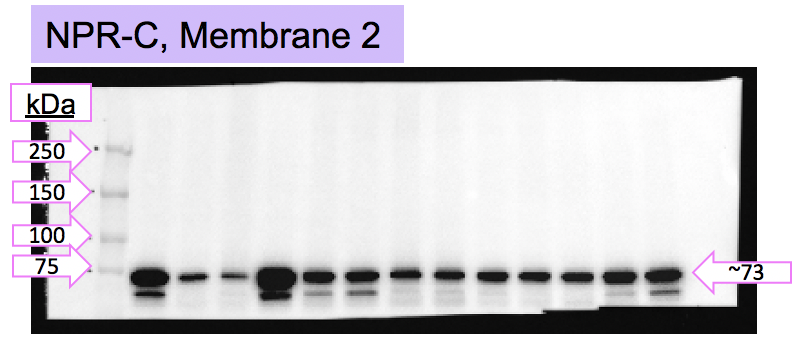

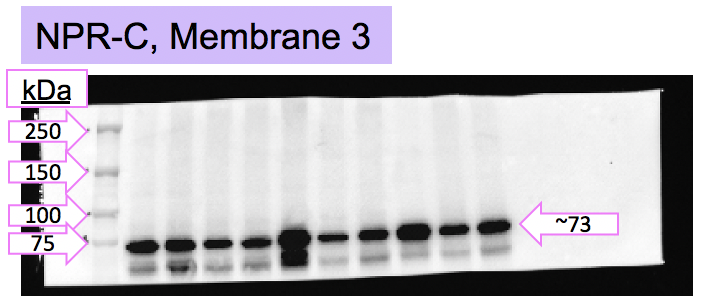


**D E F**


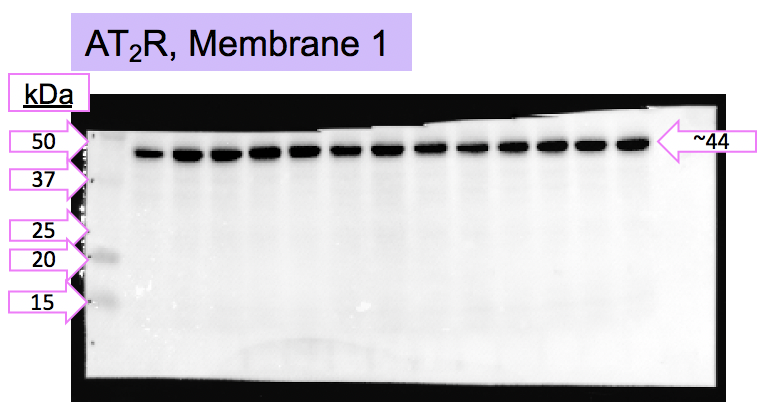

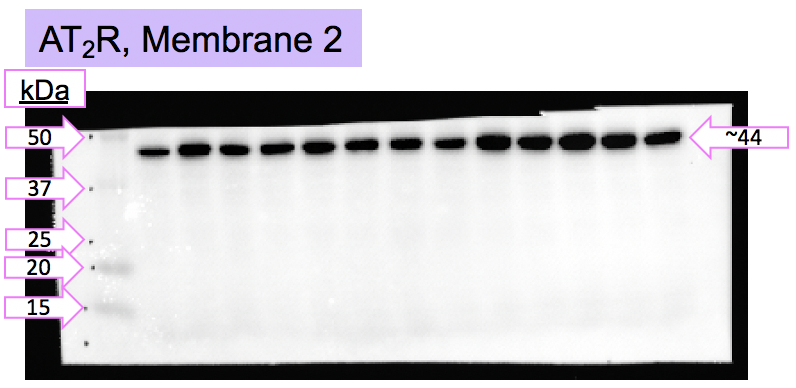

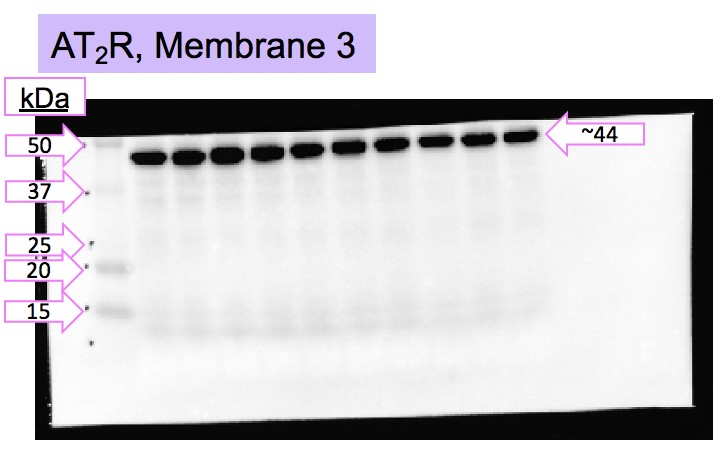


**G H I**


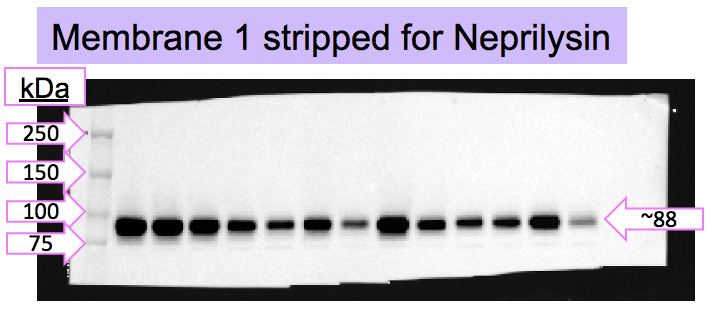

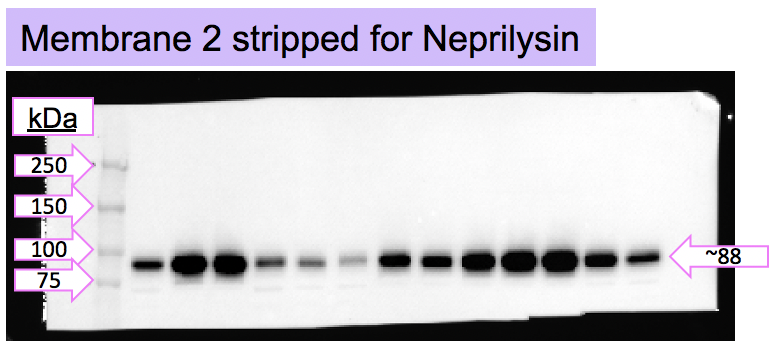

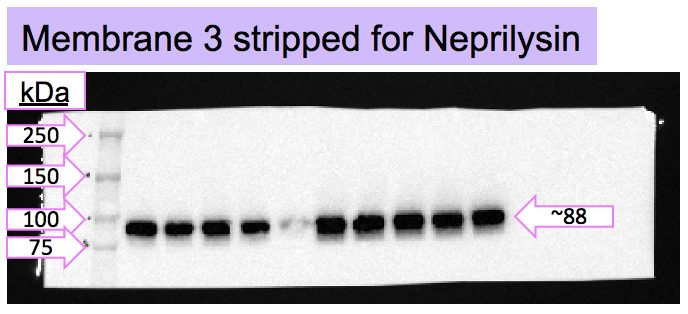


**J K L**


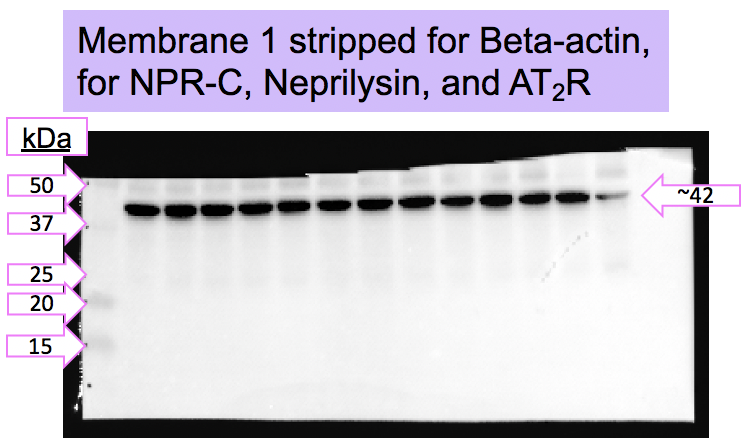

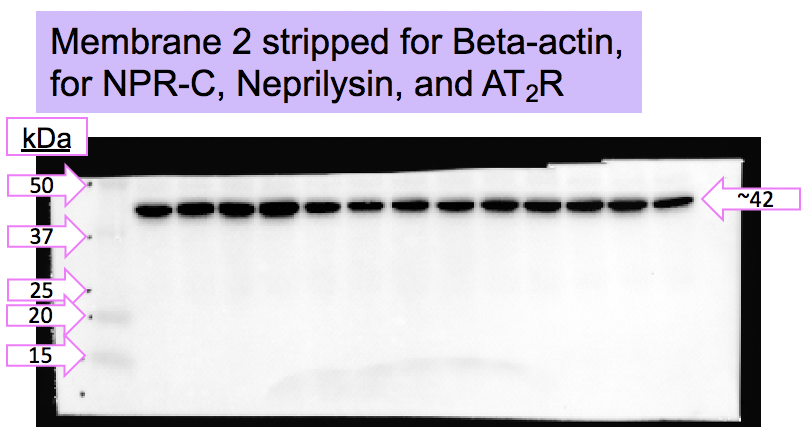

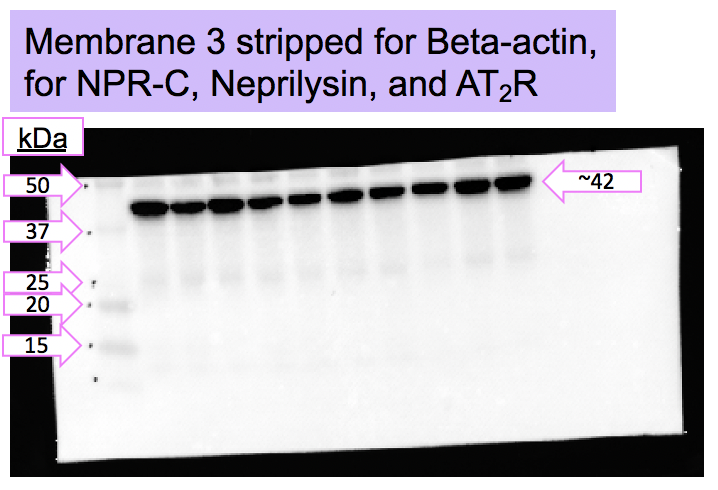


Supplementary Figure 3: Western blot membranes of renal NPR-C (A, B & C), renal AT_2_R (D, E, & F), renal neprilysin (G, H, & I), and loading control beta-actin (J, K, & L). Protein ladder was loaded into lane 1 of all three membranes, NSD loaded into lanes 3, 4 & 5 of membranes 1 & 2, and lanes 4 & 5 of membrane 3, HSD loaded into lanes 6, 7, & 8 of membranes 1 & 2, and lanes 6 & 7 of membrane 3, SAC/C21 into lanes 9, 10, & 11 of membranes 1 & 2, and lanes 8 & 9 of membrane 3, SAC/VAL into lanes 12, 13, & 14 of membranes 1 & 2, and lanes 10 & 11 of membrane 3.

**A B**
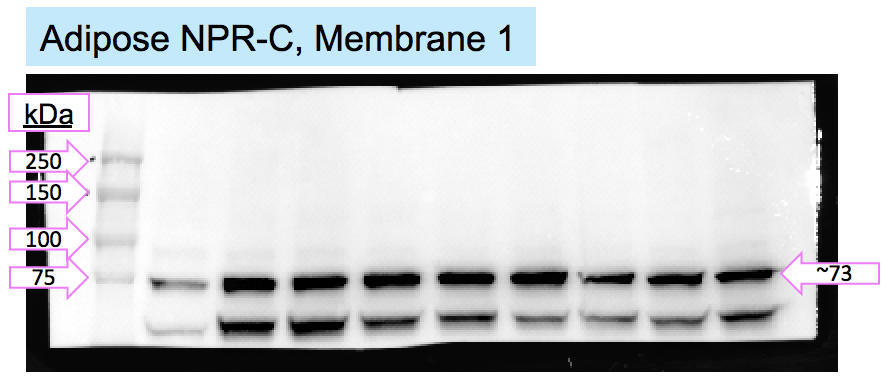

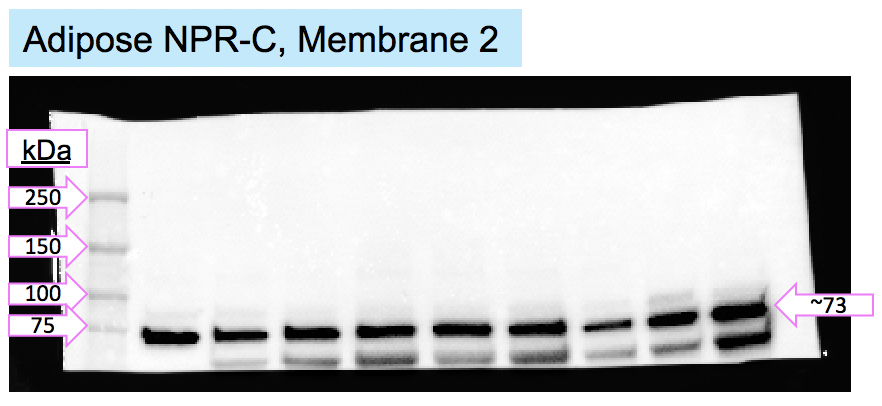


**C D**


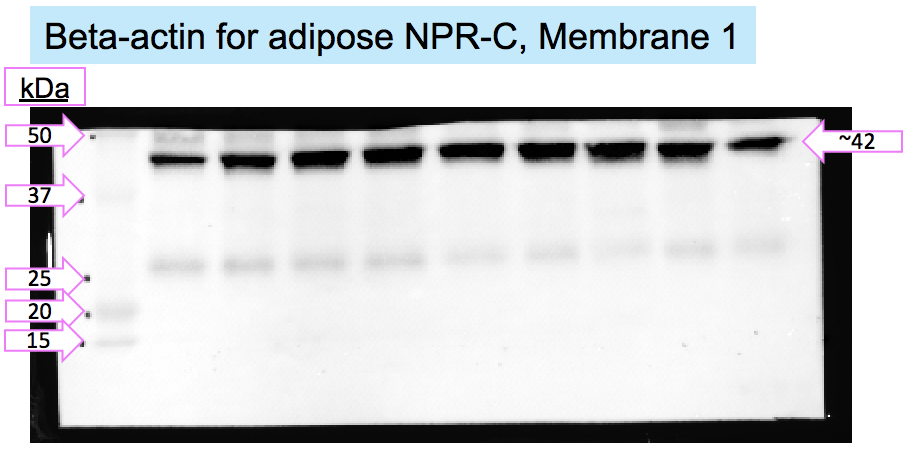

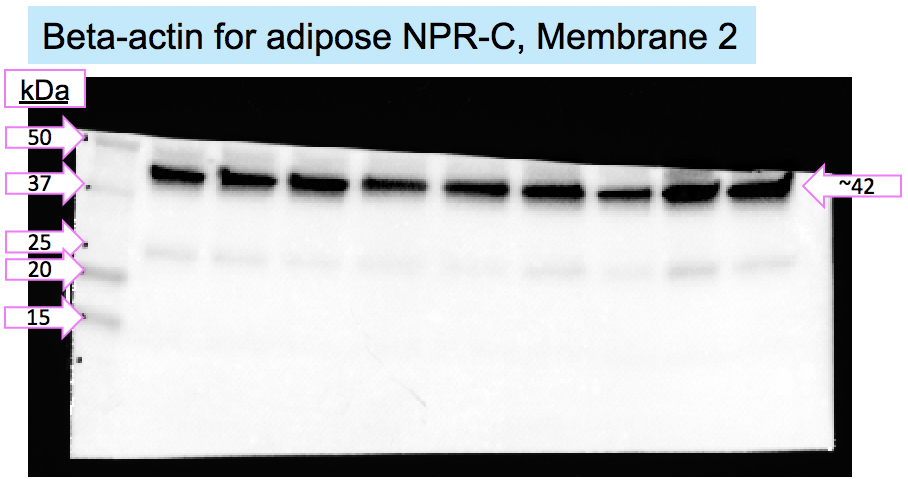


**E F**


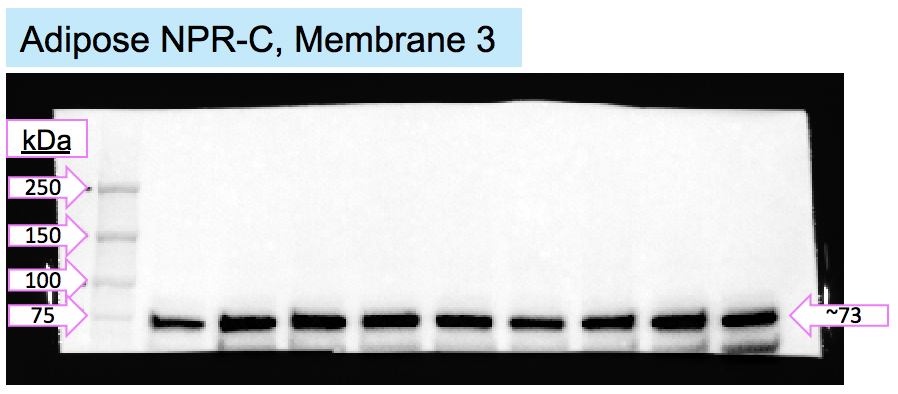

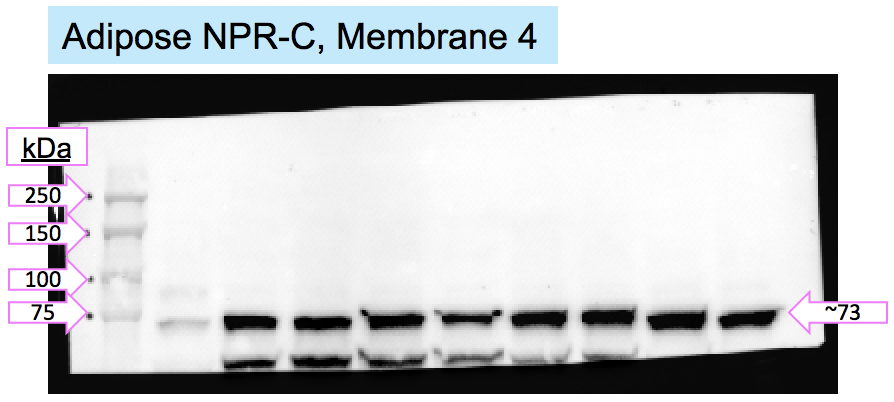


**G H**


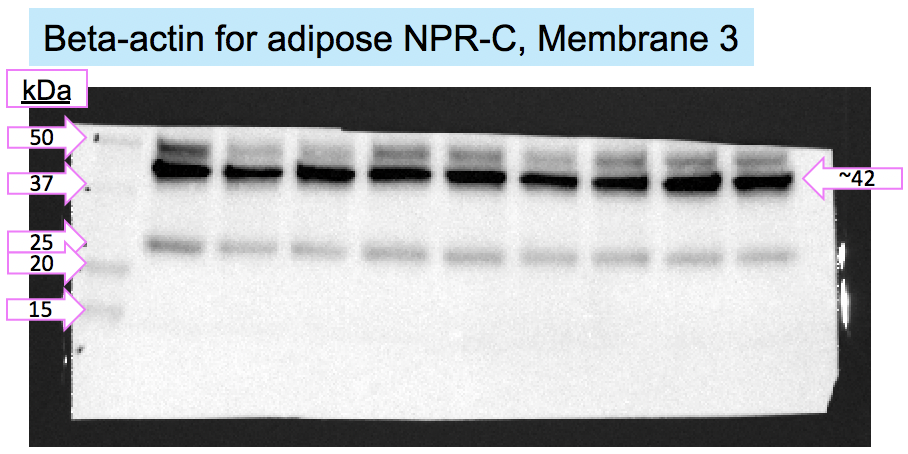

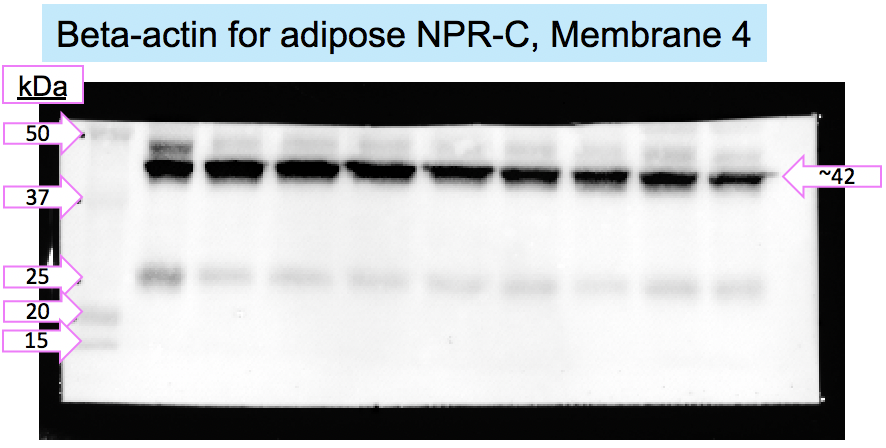


Supplementary Figure 4: Western blot membranes of white adipose tissue NPR-C (A, B, E, & F), and loading control beta-actin (C, D, G, & H). Protein ladder was loaded into lane 1 of all three membranes, NSD loaded into lanes 3, & 4 of all membranes, HSD loaded into lanes 5 & 6 of all membranes, SAC/C21 into lanes 7 & 8 of all membranes, SAC/VAL into lanes 9 & 10 of all membranes.

**A B C**
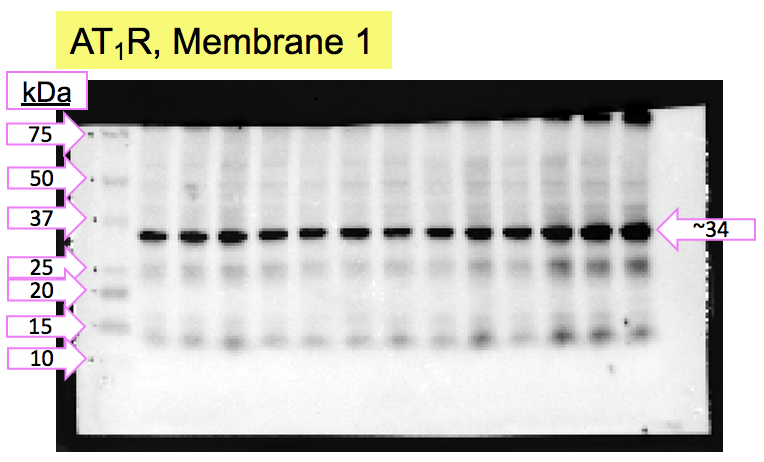

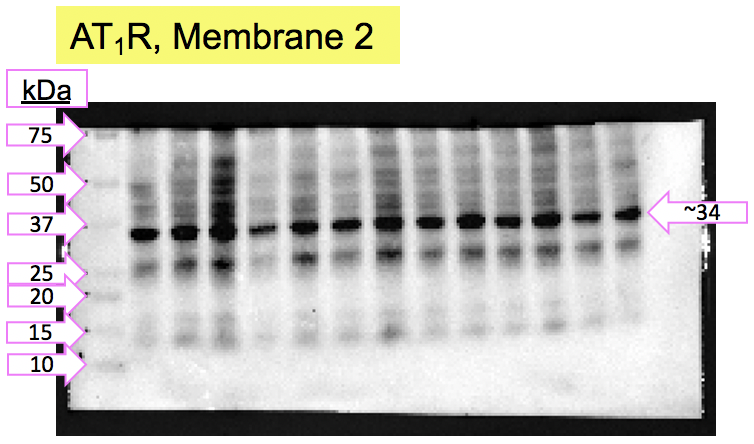

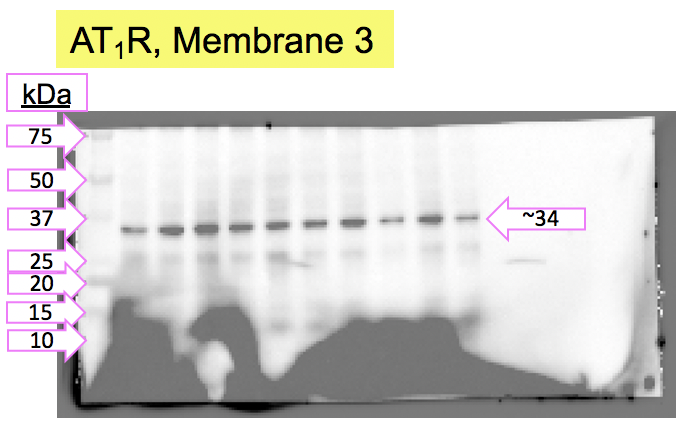


**D E F**


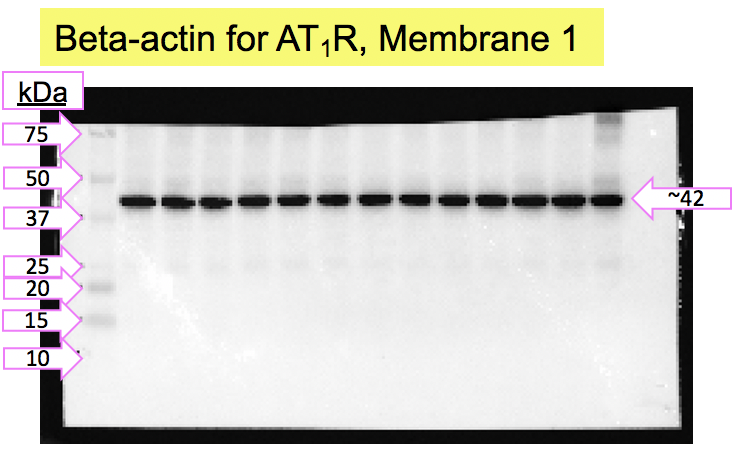

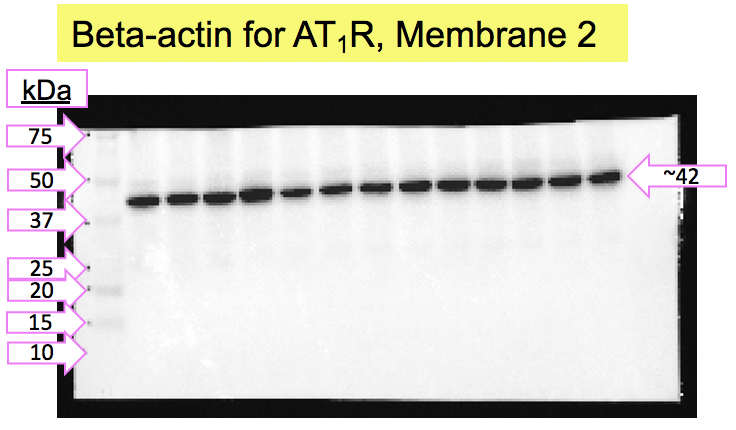

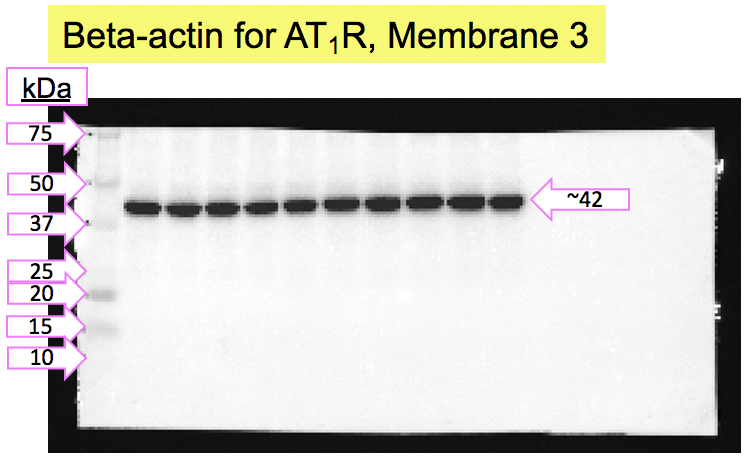


Supplementary Figure 5: Western blot membranes of renal AT_1_R (A, B & C), and loading control beta-actin (D, E, & F). Protein ladder was loaded into lane 1 of all three membranes, NSD loaded into lanes 3, 4 & 5 of membranes 1 & 2, and lanes 4 & 5 of membrane 3, HSD loaded into lanes 6, 7, & 8 of membranes 1 & 2, and lanes 6 & 7 of membrane 3, SAC/C21 into lanes 9, 10, & 11 of membranes 1 & 2, and lanes 8 & 9 of membrane 3, SAC/VAL into lanes 12, 13, & 14 of membranes 1 & 2, and lanes 10 & 11 of membrane 3.

*References:*

1. WATANABE, N., KAMEI, S., OHKUBO, A., YAMANAKA, M., OHSAWA, S., MAKINO, K. & TOKUDA, K. 1986. Urinary protein as measured with a pyrogallol red-molybdate complex, manually and in a Hitachi 726 automated analyzer. *Clinical chemistry,* 32**,** 1551-1554. [doi.org/10.1093/clinchem/32.8.1551](https://doi.org/10.1093/clinchem/32.8.1551)
2. YUEN, P. S., DUNN, S. R., MIYAJI, T., YASUDA, H., SHARMA, K. & STAR, R. A. 2004. A simplified method for HPLC determination of creatinine in mouse serum. *American Journal of Physiology-Renal Physiology,* 286**,** F1116-F1119. doi: 10.1152/ajprenal.00366.2003
